# Effects of plant active substances in rheumatoid arthritis—a systematic review and network meta-analysis

**DOI:** 10.3389/fphar.2025.1536023

**Published:** 2025-02-05

**Authors:** Qiuwei Peng, Jian Wang, Kesong Li, Congming Xia, Chuanhui Yao, Qiuyan Guo, Xun Gong, Xiaopo Tang, Quan Jiang

**Affiliations:** ^1^ Department of Rheumatology, Guang’anmen Hospital, China Academy of Chinese Medical Sciences, Beijing, China; ^2^ State Key Laboratory for Quality Ensurance and Sustainable Use of Dao-di Herbs, Artemisinin Research Center, Institute of Chinese Materia Medica, China Academy of Chinese Medical Sciences, Beijing, China

**Keywords:** rheumatoid arthritis, plant active substances, curcumin, quercetin, resveratrol, systematic review and meta-analysis

## Abstract

**Background:**

Plant active substances are extensively utilized in treating rheumatoid arthritis (RA). Despite numerous experimental and clinical studies on plant active substances their efficacy remains largely unsubstantiated. The widespread use of these extracts as therapeutic measures for RA is problematic due to the lack of compelling evidence.

**Objective:**

Our research aims to assess the impact of plant active substances on RA by conducting a network meta-analysis.

**Methods:**

We systematically searched four electronic databases—PubMed, EMBASE, the Cochrane Central Register of Controlled Trials, and Web of Science—from their inception to August 2024. The main focus was on assessing primary outcomes, including the Visual Analogue Scale (VAS), inflammatory markers, Swollen Joint Count (SJC), Tender Joint Count (TJC), and Disease Activity Score on 28 joints (DAS28). We performed data analysis using StataMP 15.1 software and ranked the therapeutic effects based on the Surface Under the Cumulative Ranking Curve (SUCRA) probability values.

**Results:**

Based on screening procedures, 18 eligible studies were incorporated into the analysis. These studies encompassed a total of 1,674 RA patients and investigated 10 different plant active substance therapies. Specifically, 10 studies included VAS indicators, 17 studies included inflammatory marker indicators, 14 studies included DAS28 indicators, 13 studies included SJC indicators, and 13 studies included TJC indicators. Based on SUCRA values, quercetin appeared to be the most effective treatment for decreasing serum VAS levels (67.3%). Furthermore, curcumin emerged as the most promising option for reducing inflammatory marker levels (72.3%), SJC (75.6%), and TJC (76.2%). Lastly, with respect to DAS28, resveratrol emerged as the optimal choice (74.3%).

**Conclusion:**

According to the network meta-analysis (NMA), curcumin exhibited superior efficacy compared to placebo in decreasing SJC and TJC. Additionally, curcumin demonstrated greater effectiveness in reducing inflammatory markers. Quercetin was more effective in reducing VAS, and resveratrol was more effective in reducing DAS28. Patients with RA may benefit from these findings. Insightful information from this study is helpful for RA patients to consider using plant active substance therapies. For their efficacy and safety to be confirmed, more proof is needed.

## Introduction

Rheumatoid arthritis (RA) is a serious autoimmune disorder where the immune system attacks joint linings, causing inflammation, joint damage, and deformity (Myasoedova et al., 2010). RA significantly impacts patients’ physical, social, and emotional health, as well as their work capacity, due to its disabling and recurrent nature (Radner et al., 2011). The etiology of RA remains unknown. The primary aims of RA treatment are to slow disease progression, minimize pain, maintain joint mobility, and prevent disability (Burmester and Pope, 2017; Dong et al., 2021). The primary treatments for RA include chemical drugs such as NSAIDs, DMARDs, and glucocorticoids (Smolen et al., 2016). Although these drugs significantly alleviate symptoms, they are costly, have numerous side effects, and yield poor long-term results (Safiri et al., 2019). The increasing number of RA patients and their economic impact are driving interest in finding safe and effective alternative treatments (Safiri et al., 2019).

Plant active substances are gaining attention for their efficacy, multi-targeting properties, and fewer side effects, and have been utilized in traditional medicine to treat RA for centuries (Chatterjee et al., 2024; Huang et al., 2024). *Tripterygium wilfordii* root extracts (TwRE) have been shown therapeutic promise in RA. A randomized controlled trial indicates that TwRE alone is as effective as methotrexate (MTX) alone for active RA, and a combination of TwRE and MTX is more effective than MTX alone (Lv et al., 2015). In a 24-week, open-label, randomized multicenter clinical trial, the results demonstrated that total glucosides of paeony (TGP) had a hepatoprotective effect in treating active RA,when combined with MTX (Chen et al., 2013). Plant active substances also exhibit anti-rheumatoid arthritis effects through diverse mechanisms. For example, resveratrol inhibited TNF-α induced production of IL-1β and MMP-3 via inhibition of PI3K-Akt signaling to possess its anti-inflammatory role in RA (Tian et al., 2013). Pomegranate extract plays a crucial role in preventing cartilage degradation by inhibiting the activation of mitogen kinase-3 (MKK3), p38α mitogen-activated protein kinase (p38α-MAPK), and runt-related transcription factor-2 (RUNX-2) (Rasheed et al., 2010). Sinomenine significantly reduced arthritic scores by modulating TNF-α, IL-6 and IL-10 levels in collagen-induced arthritis rats (Tong et al., 2015). The studies summarized provide a brief overview of the mechanisms by which plant active substances inhibit inflammation and modulate immunity.

Numerous randomized controlled trials (RCTs) studying the therapeutic effects of plant active substances on RA have been performed and published. Nevertheless, the outcomes and therapeutic interventions of these RCTs are inconsistent and do not provide a solid foundation for clinicians to develop treatment strategies for RA. Consequently, there is an urgent need for a thorough and comprehensive synthesis of these RCTs to refine the treatment paradigm for RA. Network meta-analysis is a data-driven method that combines direct and indirect evidence to compare and rank the effectiveness of multiple treatments for a condition (Rouse et al., 2017). Accordingly, this research represents the first comprehensive network meta-analysis of RCTs concerning plant active substances for RA, aiming to provide clinicians with high-quality evidence.

## Methods

The meta-analysis and systematic review were rigorously conducted in strict adherence to the Preferred Reporting Items for Systematic Reviews and Meta-Analyses (PRISMA) guidelines (Table 1), which has been registered with PROSPERO (CRD42024569765).

**TABLE 1:** Characteristics of the studies included in the meta-analysis.

Author	Country	Year	N	Patient age (Year) (Mean (SD))	Intervention(I) T C		Intervention time (Weeks)	Intervention frequency (T)	Outcome indicator(O)
Amalraj et al. (2017)	India	2017	24	T: 38.3 (5.8) C: 39.6 (8.8)	curcumin	placebo	12W	Bid	①②③④⑤
Chandran and Goel (2012)	India	2012	26	T: 47.8 (8.60) C: 47 (16.22)	curcumin	CON	8W	Bid	①②③④⑤
Pourhabibi-Zarandi et al. (2022)	Iran	2022	44	T: 50.68 (9.93) C: 50.36 (9.70)	curcumin	placebo	8W	500 mg/day	②
Pourhabibi-Zarandi et al. (2024)	Iran	2024	44	T: 50.68 (9.93) C: 50.36 (9.70)	curcumin	placebo	8W	500 mg/day	①③④⑤
Rezaieyazdi et al. (2023)	Iran	2023	61	T: 55 (12.25)	curcumin	placebo	12W	80 mg/day	①②③④⑤
Hemmati (Hemmati et al., 2016)	Iran	2016	60	NR	curcumin	placebo	8W	Qd	②③④⑤
Bitler (Bider et al., 2007)	United States	2007	27	T + C: 65 (5)	olive extract	placebo	8W	Bid	②
Chen et al. (2012)	China	2012	194	T + C: 44.6 (13.3)	TGP	placebo	24W	Tid	②③
Xiang et al. (2015)	China	2015	268	T: 49.57 (12.57) C: 47.57 (11.68)	TGP	CON	12W	Tid	②③④⑤
Ghavipour et al. (2017)	Iran	2017	55	T: 48.4 (11.4) C: 49.1 (12.2)	pomegranate extract	placebo	8W	Qd	①②③④⑤
Goldbach-Mansky et al. (2009)	United States	2009	62	T: 54 (11) C: 52 (12)	TwRE	CON	24W	Tid	①②④⑤
Lv et al. (2015)	China	2015	138	T: 51.3 (8.3) C: 51.0 (10.3)	TwRE	CON	24W	Tid	①②③④⑤
Hang et al. (2018)	China	2018	331	T: 51.3 (4.2) C: 52.1 (5.8)	baicalin	placebo	12W	Qd	②
Helli et al. (2019)	Iran	2019	44	T + C: 55.49 (5.98)	sesamin	placebo	6W	Qd	①②③④⑤
Javadi et al. (2017)	Iran	2017	40	T: 46.55 (9.94) C: 48.00 (8.39)	quercetin	placebo	8W	500 mg/day	①②③④⑤
Khojah et al. (2018)	Saudi Arabia	2018	100	T: 44.2 (16.4) C: 46.5 (12.3)	resveratrol	placebo	12W	Qd	②③④⑤
Shi et al. (2022)	China	2022	77	NR	SIN	CON	24W	Bid	①②③④⑤
Yang et al. (2018)	China	2018	79	T: 52.97 (9.96) C:54.05 (14.89)	puerarin	placebo	24W	400 mg/day	②③

Note: T, experimental group, C, control group, CON, control group with usual treatment, NR, not reported, TGP, total glucosides of paeony, TwRE, *tripterygium wilfordii* root extract, SIN, sinomenine; ① pain visual analog scale (VAS) ② inflammatory marker ③ Disease Activity Score 28 (DAS28) ④ swollen joint count (SJC) ⑤ tender joint count (TJC).

### Search strategy

In this study, an exhaustive literature review was performed utilizing four electronic databases—PubMed, EMBASE, the Cochrane Central Register of Controlled Trials, and Web of Science—spanning from their inception to August 2024. The search strategy was formulated in accordance with the PICOS framework: (P) Population: individuals with RA; (I) Intervention: plant active substances; (C) Comparator: control group receiving only usual care or placebo; (O) Outcomes: Disease Activity Score on a 28-joint count (DAS28), swollen joint count (SJC), tender joint count (TJC), inflammatory markers including C-reactive protein (CRP) and Erythrocyte Sedimentation Rate (ESR), and Visual Analogue Scale (VAS) for pain; (S) Study type: RCTs. The detailed search strategy is provided in Supplementary Table 1.

### Inclusion criteria

Included studies were randomized controlled trials that reported outcome metrics comparing treatment and control groups in adults with RA treated with plant active substances. Studies that did not meet the inclusion criteria, or that did not exclusively target RA patients (e.g., those including patients with osteoarthritis), were excluded. Uncontrolled and/or non-randomized studies, pediatric studies and review articles were also excluded.

### Exclusion criteria

We excluded research characterized by incomplete or undocumented data, as well as studies involving non-randomized controlled trials, including quasi-randomized controlled trials, study protocols, animal experiments, case studies, conference abstracts, and letters to the editor.

### Study selection

Using Zotero software, we systematically filtered and excluded literature. Initially, two researchers independently assessed the titles to identify and remove duplicates, non-randomized studies, reviews, protocols, conference papers and correspondence. Subsequently, they examined the abstracts to decide which studies to include or exclude. The final phase involved a comprehensive review of the selected literature to make definitive inclusion decisions. Throughout this process, the researchers independently screened the literature and subsequently compared their lists to ensure concordance. In cases of disagreement, a consensus was reached through discussion. When their selections aligned, the literature progressed to the final review stage. When facing discrepancies, a third researcher facilitated discussions to reconcile the differences.

### Data extraction

The following headings will be captured in a six-item standardized and pre-selected data extraction form: (1) author, (2) country, (3) year of publication, (4) mean patient age, (5) details of the plant active substance intervention, and (6) outcome indicators.

### Risk of bias of individual studies

Two researchers independently evaluated the risk of bias (ROB) in RCTs using the Cochrane Handbook version 5.1.0. We conducted a systematic evaluation of seven critical aspects of the studies: (1) the randomization method, (2) allocation concealment, (3) participant blinding, (4) personnel blinding, (5) the management of missing data, (6) the comprehensiveness of reported outcomes, and (7) other sources of bias. Based on this evaluation, studies were classified into three levels of risk of bias: high (with five or more domains at high risk), moderate (three or four domains at high risk), and low (no more than two domains at high risk) (Higgins et al., 2011).

### Quality assessment of evidence

We used the CINeMA (Confidence in Network Meta-Analysis) framework to assess the quality of assessment (Nikolakopoulou et al., 2020; Papakonstantinou et al., 2020). It includes six key dimensions: including within-study bias, reporting bias, indirectness, imprecision, heterogeneity, and inconsistency.

### Data analysis

The variables in plant active substance studies are continuous and are expressed as means with standard deviations (SD) (Li and Chen, 2021). To address the non-uniformity of units for outcome variables across some studies, continuous variables were recalculated using the 95% confidence interval (CI) and standardized mean difference (SMD). I^2^ statistic test was performed, the random effects model was used when I^2^ >50% and P < 0.05, and the fixed effects model was used for the meta-analysis. Given the potential differences among the studies, we opted to use a random effects model for the analysis (Jackson et al., 2011). The details of I^2^ statistic test are provided in Supplementary Figures 1–5. We used Stata software (version 15.1) for our network meta-analysis, employing a Bayesian approach with Markov chain Monte Carlo simulations. Our methodology followed PRISMA NMA guidelines (Supplementary Material 2) to ensure transparency, reproducibility, and rigor (Moher et al., 2015; Vats et al., 2019). We used Node-splitting method in Stata to assess indirect and direct comparisons consistency in our analysis, with P-value above 0.05 indicating consistency (Salanti et al., 2011).

Stata software is employed to construct network diagrams for the evaluation of plant active substance interventions. In these diagrams, nodes symbolize individual interventions or control groups, while the connecting lines denote direct comparisons between them. The dimensions of the nodes and lines are positively correlated with the number of studies (Chaimani et al., 2013). We evaluated the ranking of each intervention by calculating the surface under the cumulative ranking curve (SUCRA) values. Interpretation of the SUCRA, which are expressed as percentages, necessitates caution unless clinical significance is evident, thereby guiding the evaluation of the efficacy of plant active substance therapies (Marotta et al., 2020). In addition, in order to assess the potential impact of small-scale research bias on NMA results, network funnel plots were drawn and examined for symmetry (Khera et al., 2016).

## Results

### Study and identification and selection

**FIGURE 1:**
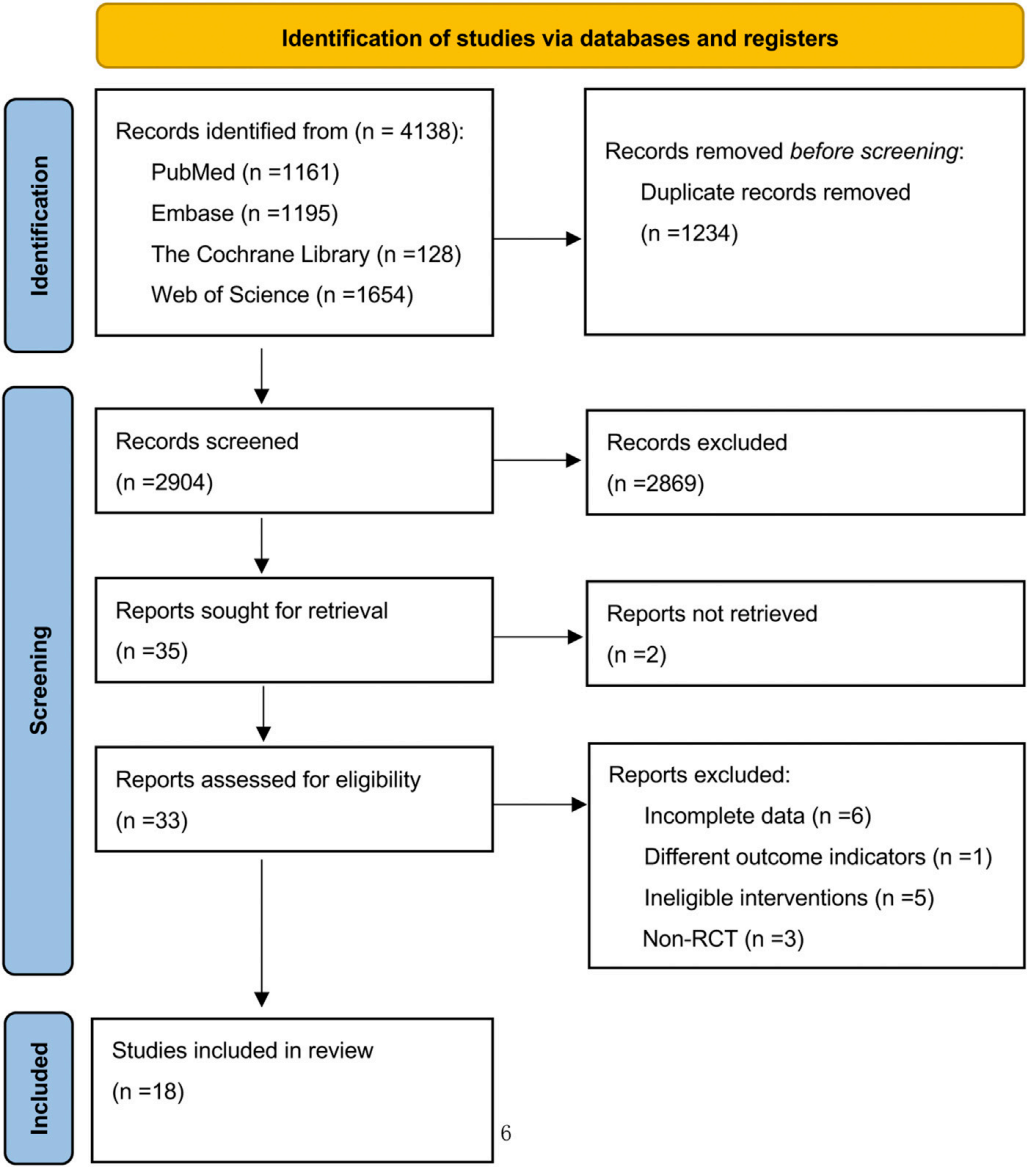
Preferred reporting items for systematic reviews and meta-analysis (PRISMA) flow diagram of systematic search and included studies.

In our electronic database retrieval process, a total of 4,138 pieces of documents were obtained. After de-duplication, the 2,904 remaining documents were reviewed for title and abstract, and then 2,869 documents were excluded. The remaining 35 documents were thoroughly reviewed; however, 2 documents were not retrieved. Fifteen documents were excluded again for various reasons, such as incomplete data, differing outcome indicators, inclusion of ineligible interventions, and non-randomized controlled trials. Consequently, the final number of documents included in this study was 18 (Figure 1).

### Characteristic and quality of the included studies

**FIGURE 2:**
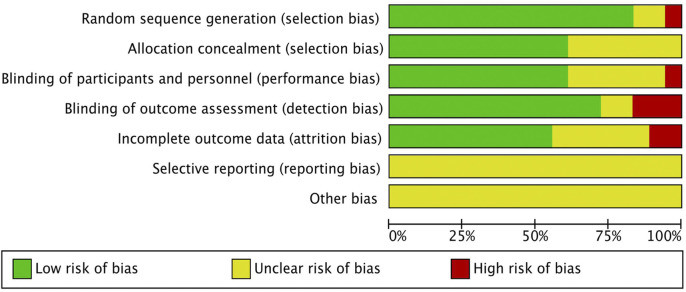
Risk of bias graph for RCTs.

Our analysis included 18 randomized controlled trials, encompassing 1,674 patients. Interventions in the treatment group included curcumin (6 studies), olive extract (1 study), TGP (2 studies), pomegranate extract (1 study), TwRE (2 studies), baicalin (1 study), sesamin (1 study), quercetin (1 study), resveratrol (1 study), sinomenine (SIN) (1 study), and puerarin (1 study). Ten studies utilized VAS as an outcome indicator, seventeen studies reported inflammatory markers, fourteen studies reported DAS28, thirteen studies reported SJC, and thirteen studies reported TJC. Two studies were from India, seven from Iran, two from the Americas, six from China, and one from Saudi Arabia. The characteristics of the included studies are shown in Table 1. The risk of bias assessment for the included RCTs is illustrated in Figure 2. After assessing the level of evidence using CINeMA, overall quality of VAS, inflammatory markers, DAS28, SJC and TJC were high confidence (Supplementary Tables 2–6).

### Pain visual analog scale

**FIGURE 3:**
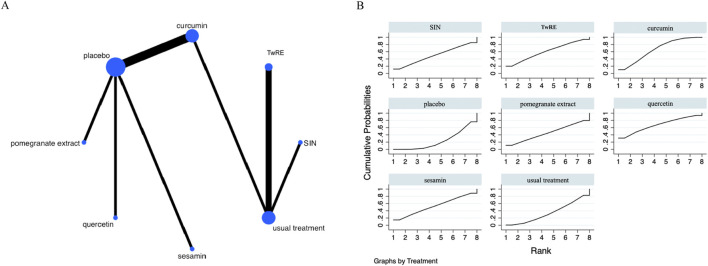
Evidence of plant active substance to influence VAS **(A)**, NMA figure of the VAS **(B)**, the SUCRA plot of the VAS. Abbreviations: TwRE, *Tripterygium wilfordii* root extract; SIN, sinomenine.

The Network Meta-Analysis figure for VAS is presented in Figure 3A. P-values were systematically evaluated for consistency across all comparative analyses within the study, encompassing both indirect and direct assessments. Since all P-values exceeded 0.05, this suggests an acceptable level of consistency among the studies. Further details are provided in Supplementary Table 7.

The findings of the Network Meta-Analysis indicated that none of the plant active substance treatments demonstrated superior efficacy compared to the control group in decreasing VAS scores. The efficacy of different plant active substance treatments in terms of lowering VAS scores was ranked, with quercetin (SUCRA: 67.3%, as shown in Figure 3B) being identified as the most effective. A comparative analysis of the different interventions is provided in Table 2.

**TABLE 2:** Treatments ranked by SUCRA values and the league table of their relative effectiveness in terms of VAS.

Quercetin SUCRA 67.3%							
−2.78 (−38.61, 33.06)	Curcumin SUCRA 66.2%						
−4.62 (−54.66, 45.43)	−1.84 (−37.67, 33.99)	TwRE SUCRA 60.7%					
−7.53 (−51.20, 36.14)	−4.75 (−39.10, 29.59)	−2.91 (−51.90, 46.08)	Sesamin SUCRA 52%				
−9.02 (−59.54, 41.49)	−6.25 (−43.45, 30.95)	−4.40 (−38.14, 29.33)	−1.49 (−50.97, 47.98)	SIN SUCRA 49.5%			
−11.10 (−56.21, 34.01)	−8.32 (−44.47, 27.83)	−6.48 (−56.75, 43.79)	−3.57 (−47.50, 40.36)	−2.08 (−52.82, 48.67)	Pomegranate extract SUCRA 45.8%		
−14.62 (−60.22, 30.98)	−11.84 (−41.15, 17.46)	−10.00 (−30.68, 10.67)	−7.09 (−51.53, 37.35)	−5.60 (−32.57, 21.37)	−3.52 (−49.37, 42.33)	Usual treatment SUCRA 35%	
−18.90 (−50.62, 12.81)	−16.13 (−32.95, 0.70)	−14.28 (−53.26, 24.69)	−11.37 (−41.40, 18.65)	−9.88 (−49.62, 29.86)	−7.80 (−39.88, 24.27)	−4.28 (−37.35, 28.79)	Placebo SUCRA 23.5%

Abbreviations: TwRE, *Tripterygium wilfordii* root extract; SIN, sinomenine.

### Inflammatory marker

**FIGURE 4:**
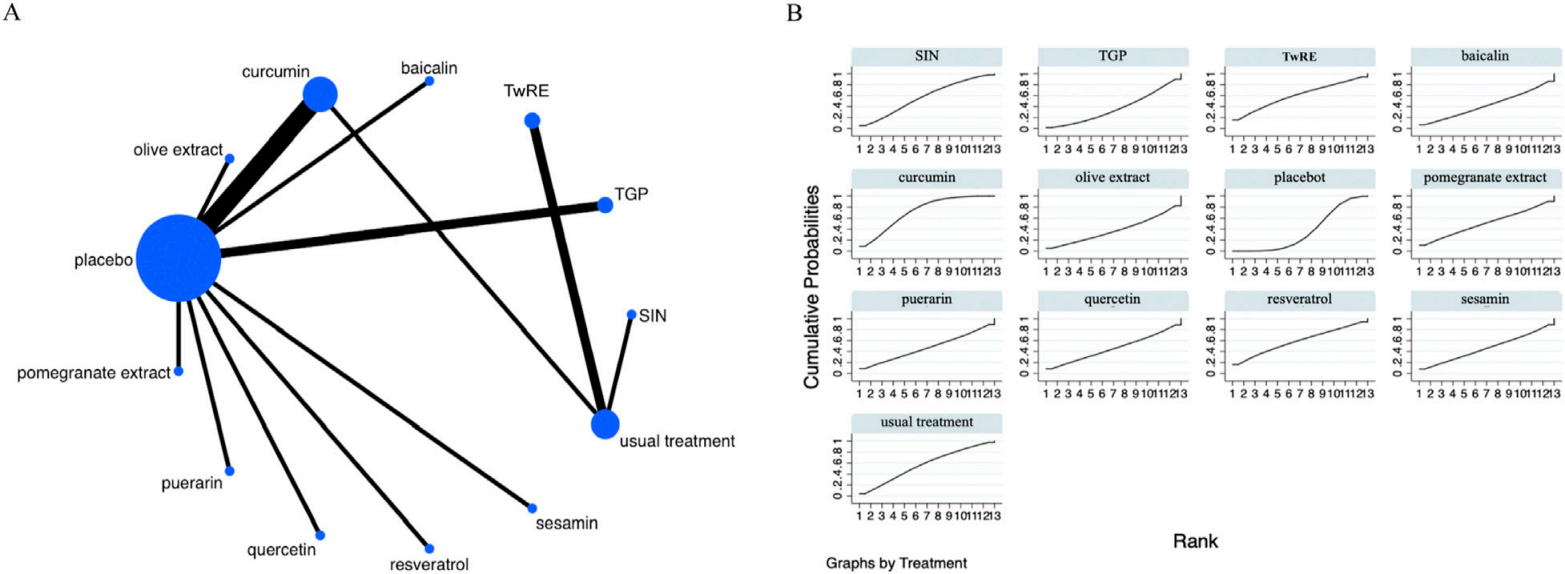
Evidence that plant active substances affect inflammatory markers **(A)**, NMA figure of the inflammatory marker **(B)**, the SUCRA plot of the inflammatory marker. Abbreviations: TwRE, *Tripterygium wilfordii* root extract; SIN, sinomenine; TGP, total glucosides of paeony.

The Network Meta-Analysis figure for inflammatory markers is presented in Figure 4A. The consistency and inconsistency between indirect and direct study comparisons were assessed. All resultant P-values were larger than 0.05, indicating an acceptable level of consistency. Further fully details are provided in Supplementary Table 8.

The Network Meta-Analysis showed that none of the plant active substance treatments outperformed the routine measures in lowering inflammatory markers. Curcumin (SUCRA: 72.3%, Figure 4B) was shown to be the most effective plant active substance intervention in terms of reducing inflammatory markers based on a probability ranking. A comparison of the different interventions is provided in Table 3.

**TABLE 3:** Treatments ranked by SUCRA values and the league table of their relative effectiveness in terms of inflammatory markers.

Curcumin SUCRA 72.3%												
−6.53 (−74.92, 61.85)	TwRE SUCRA 60.2%											
−8.34 (−79.42, 62.75)	−1.80 (−96.48, 92.87)	Resveratrol SUCRA 58.8%										
−10.78 (−61.48, 39.92)	−4.24 (−69.20, 60.71)	−2.44 (−81.92, 77.04)	SIN SUCRA 57.2%									
−10.23 (−62.66, 42.20)	−3.69 (−49.02, 41.64)	−1.89 (−85.93, 82.15)	0.55 (−48.47, 49.58)	Usual treatment SUCRA 56.8%								
−17.14 (−89.68, 55.40)	−10.60 (−106.37, 85.17)	−8.80 (−100.64, 83.04)	−6.36 (−87.14, 74.43)	−6.91 (−92.19, 78.37)	pomegranate extract SUCRA 50.9%							
−21.11 (−92.56, 50.34)	−14.57 (−109.52, 80.38)	−12.77 (−103.76, 78.22)	−10.33 (−90.14, 69.48)	−10.88 (−95.23, 73.47)	−3.97 (−96.10, 88.16)	Puerarin SUCRA 47%						
−21.96 (−92.86, 48.95)	−15.42 (−109.96, 79.11)	−13.62 (−104.17, 76.93)	−11.18 (−90.50, 68.14)	−11.73 (−95.62, 72.16)	−4.82 (−96.52, 86.88)	−0.85 (−91.69, 89.99)	Sesamin SUCRA 46.3%					
−21.44 (−92.78, 49.91)	−14.90 (−109.77, 79.97)	−13.10 (−104.00, 77.80)	−10.66 (−90.37, 69.06)	−11.21 (−95.47, 73.05)	−4.30 (−96.35, 87.75)	−0.33 (−91.52, 90.86)	0.52 (−90.24, 91.28)	Quercetin SUCRA 46%				
−24.39 (−95.19, 46.42)	−17.85 (−112.31, 76.61)	−16.05 (−106.53, 74.43)	−13.61 (−92.83, 65.62)	−14.16 (−97.96, 69.64)	−7.25 (−98.88, 84.38)	−3.28 (−94.05, 87.49)	−2.43 (−92.77, 87.91)	−2.95 (−93.64, 87.74)	Baicalin SUCRA 43.1%			
−29.68 (−100.64, 41.27)	−23.15 (−117.73, 71.43)	−21.35 (−111.94, 69.25)	−18.90 (−98.26, 60.46)	−19.46 (−103.39, 64.47)	−12.55 (−104.29, 79.20)	−8.58 (−99.46, 82.31)	−7.73 (−98.18, 82.73)	−8.25 (−99.05, 82.56)	−5.30 (−95.68, 85.09)	olive extract SUCRA 38.2%		
−29.16 (−83.90, 25.57)	−22.63 (−105.72, 60.47)	−20.82 (−99.37, 57.72)	−18.38 (−83.63, 46.86)	−18.93 (−89.68, 51.81)	−12.02 (−91.89, 67.84)	−8.05 (−86.93, 70.82)	−7.20 (−85.58, 71.18)	−7.72 (−86.51, 71.06)	−4.77 (−83.07, 73.52)	0.52 (−77.91, 78.95)	TGP SUCRA 37.1%	
−27.35 (−58.54, 3.84)	−20.81 (−91.49, 49.87)	−19.01 (−83.19, 45.17)	−16.57 (−65.86, 32.72)	−17.12 (−72.72,38.48)	−10.21 (−76.00, 55.58)	−6.24 (−70.83, 58.34)	−5.39 (−69.37, 58.59)	−5.91 (−70.38, 58.56)	−2.96 (−66.83, 60.91)	2.33 (−61.71, 66.38)	1.81 (−43.60, 47.23)	Placebo SUCRA 36.1%

Abbreviations: TwRE, *Tripterygium wilfordii* root extract; SIN, sinomenine; TGP, total glucosides of paeony.

### Disease Activity Score 28

**FIGURE 5:**
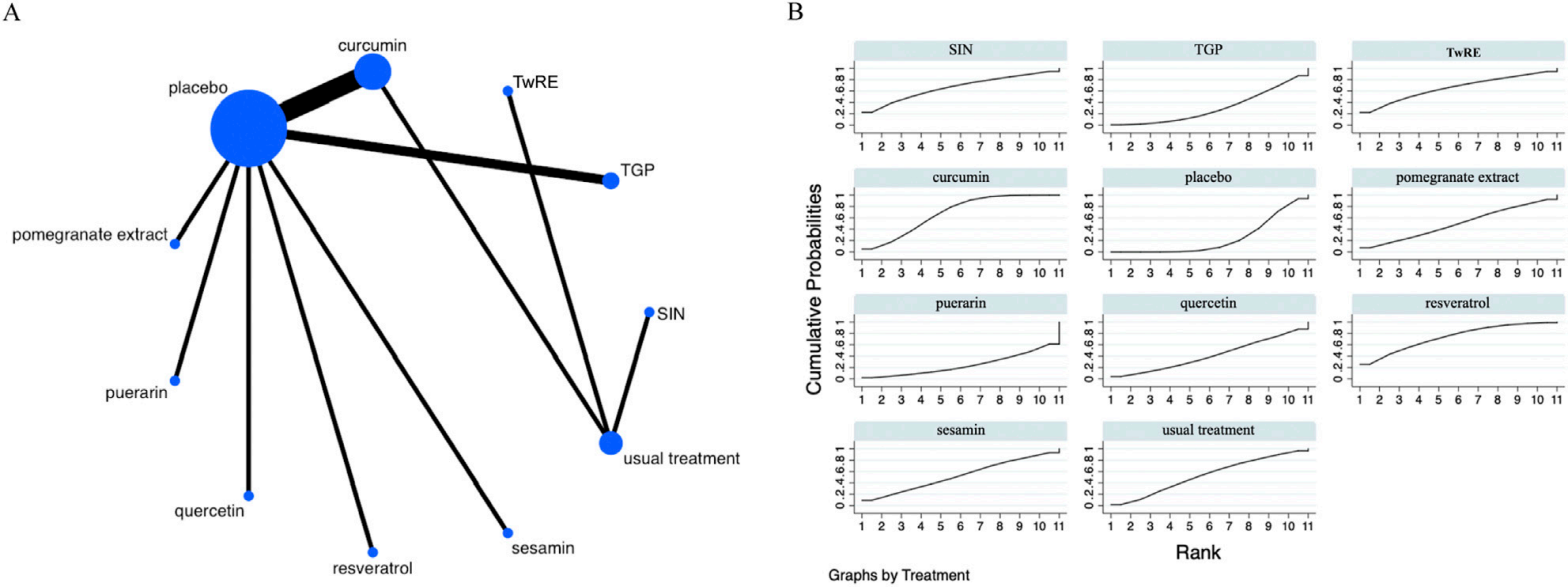
Evidence that plant active substances affect DAS28 **(A)**, NMA figure of the DAS28 **(B)**, the SUCRA plot of the DAS28. Abbreviations: TwRE, *Tripterygium wilfordii* root extract; SIN, sinomenine; TGP, total glucosides of paeony.

The Network Meta-Analysis figure for DAS28 is displayed in Figure 5A. Consistency and inconsistency were tested for all indirect and direct comparisons, all p-values were found to be greater than 0.05, which indicates a reasonable level of consistency among the studies. Further fully details are provided in Supplementary Table 9.

The Network Meta-Analysis results indicated that, compared to the placebo, curcumin [MD = −1.33, 95% CI (−2.30, −0.36)] was superior in decreasing DAS28. The probability ranking of various plant active substance interventions for decreasing DAS28 identified resveratrol as the highest (SUCRA: 73.3%, as shown in Figure 5B). A comprehensive comparison of the various interventions is presented in Table 4.

**TABLE 4:** Treatments ranked by SUCRA values and the league table of their relative effectiveness in terms of DAS28.

Resveratrol SUCRA 73.3%										
−0.33 (−2.49, 1.83)	Curcumin SUCRA 68.6%									
−0.17 (−3.77, 3.43)	0.16 (−2.72, 3.04)	SIN SUCRA 66.3%								
−0.21 (−3.83, 3.40)	0.12 (−2.78, 3.01)	−0.04 (−2.84, 2.76)	TwRE SUCRA 65.4%							
−0.67 (−3.69, 2.35)	−0.34 (−2.44, 1.76)	−0.50 (−2.47, 1.47)	−0.46 (−2.45, 1.53)	Usual treatment SUCRA 53.5%						
−0.78 (−3.63, 2.07)	−0.45 (−2.76, 1.86)	−0.61 (−4.30, 3.08)	−0.57 (−4.27, 3.14)	−0.11 (−3.23, 3.02)	Sesamin SUCRA 52.7%					
−0.86 (−3.64, 1.92)	−0.53 (−2.76, 1.70)	−0.69 (−4.33, 2.95)	−0.65 (−4.30, 3.00)	−0.19 (−3.25, 2.88)	−0.08 (−2.98, 2.82)	Pomegranate extract SUCRA 50%				
−1.20 (−4.01, 1.61)	−0.87 (−3.12, 1.38)	−1.03 (−4.68, 2.63)	−0.99 (−4.66, 2.68)	−0.53 (−3.61, 2.55)	−0.42 (−3.34, 2.50)	−0.34 (−3.19, 2.51)	Quercetin SUCRA 41.5%			
−1.52 (−3.89, 0.84)	−1.19 (−2.86, 0.48)	−1.35 (−4.68, 1.98)	−1.31 (−4.65, 2.04)	−0.85 (−3.54, 1.84)	−0.74 (−3.24, 1.76)	−0.66 (−3.08, 1.76)	−0.32 (−2.77, 2.13)	TGP SUCRA 30.7%		
−1.94 (−5.05, 1.17)	−1.61 (−4.24, 1.02)	−1.77 (−5.66, 2.13)	−1.73 (−5.64, 2.18)	−1.27 (−4.63, 2.10)	−1.16 (−4.38, 2.06)	−1.08 (−4.24, 2.08)	−0.74 (−3.92, 2.44)	−0.42 (−3.22, 2.38)	Puerarin SUCRA 24.2%	
−1.66 (−3.59, 0.27)	−1.33 (−2.30,-0.36)	−1.49 (−4.52, 1.55)	−1.45 (−4.50, 1.61)	−0.99 (−3.30, 1.33)	−0.88 (−2.98, 1.22)	−0.80 (−2.80, 1.20)	−0.46 (−2.49, 1.57)	−0.14 (−1.50, 1.22)	0.28 (−2.16, 2.72)	Placebo SUCRA 23.8%

Abbreviations: TwRE, *Tripterygium wilfordii* root extract; SIN, sinomenine; TGP, total glucosides of paeony.

### Swollen joint count

**FIGURE 6:**
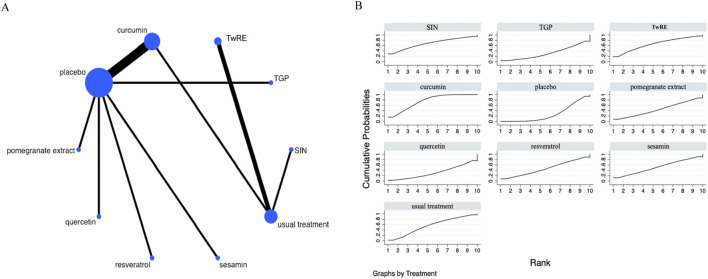
Evidence that plant active substances affect SJC **(A)**, NMA figure of the SJC **(B)**, the SUCRA plot of the SJC. Abbreviations: SIN, sinomenine; TwRE, *Tripterygium wilfordii* root extract; TGP, total glucosides of paeony.

The Network Meta-Analysis figure for swollen joint count (SJC) is presented in Figure 6A. All indirect and direct comparisons were assessed for consistency and inconsistency; all P-values exceeded 0.05, suggesting good consistency. Further fully details are provided in Supplementary Table 10.

Based on the Network Meta-Analysis results, curcumin [MD = −4.41, 95% CI (−7.50, −1.31)] was superior to the placebo in reducing SJC. The probability ranking of various plant active substance interventions for decreasing SJC identified curcumin as the highest (SUCRA: 75.6%, as shown in Figure 6B). A comprehensive comparison of the various interventions is showed in Table 5.

**TABLE 5:** Treatments ranked by SUCRA values and the league table of their relative effectiveness in terms of SJC.

Curcumin SUCRA 75.6%									
−0.02 (−8.77, 8.74)	SIN SUCRA 68.1%								
−0.53 (−8.17, 7.11)	−0.52 (−8.13, 7.10)	TwRE SUCRA 65.9%							
−1.49 (−7.72, 4.74)	−1.48 (−7.69, 4.73)	−0.96 (−5.37, 3.45)	Usual treatment SUCRA 56%						
−2.19 (−9.57, 5.18)	−2.18 (−13.61, 9.26)	−1.66 (−12.27, 8.95)	−0.70 (−10.35, 8.95)	Sesamin SUCRA 52.2%					
−2.80 (−9.67, 4.06)	−2.79 (−13.90, 8.32)	−2.27 (−12.53, 7.99)	−1.31 (−10.57, 7.95)	−0.61 (−9.68, 8.46)	Resveratrol SUCRA 46.8%				
−3.02 (−10.07, 4.02)	−3.01 (−14.23, 8.22)	−2.49 (−12.87, 7.89)	−1.53 (−10.93, 7.86)	−0.83 (−10.04, 8.38)	−0.22 (−9.03, 8.59)	Pomegranate extract SUCRA 44.7%			
−4.40 (−11.28, 2.47)	−4.39 (−15.51, 6.73)	−3.87 (−14.14, 6.39)	−2.91 (−12.18, 6.36)	−2.21 (−11.29, 6.87)	−1.60 (−10.27, 7.07)	−1.38 (−10.20, 7.44)	Quercetin SUCRA 31.9%		
−4.40 (−11.27, 2.47)	−4.39 (−15.50, 6.73)	−3.87 (−14.14, 6.39)	−2.91 (−12.18, 6.36)	−2.21 (−11.29, 6.87)	−1.60 (−10.27, 7.07)	−1.38 (−10.19, 7.43)	0.00 (−8.68, 8.68)	TGP SUCRA 31.8%	
−4.41 (−7.50,-1.31)	−4.39 (−13.66, 4.88)	−3.87 (−12.11, 4.36)	−2.91 (−9.86, 4.04)	−2.21 (−8.91, 4.48)	−1.60 (−7.73, 4.52)	−1.38 (−7.71, 4.95)	−0.00 (−6.14, 6.14)	−0.00 (−6.14, 6.13)	Placebo SUCRA 27.1%

Abbreviations: SIN, sinomenine; TwRE, *Tripterygium wilfordii* root extract; TGP, total glucosides of paeony.

### Tender joint count

**FIGURE 7:**
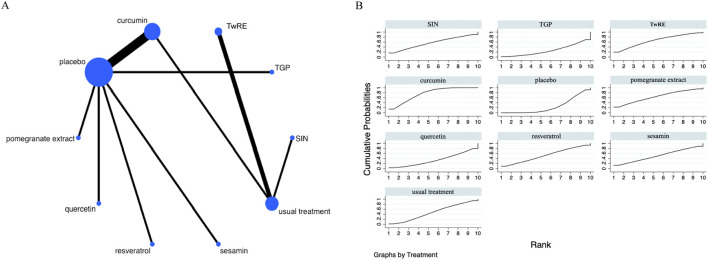
Evidence that plant active substances influence TJC **(A)**, NMA figure of the TJC **(B)**, the SUCRA plot for TJC. Abbreviations: TwRE, *Tripterygium wilfordii* root extract; SIN, sinomenine; TGP, total glucosides of paeony.

The Network Meta-Analysis figure for tender joint count (TJC) is presented in Figure 7A. Indirect and direct study comparisons were assessed for inconsistency and consistency, with all P-values exceeding 0.05, thereby indicating uniform effects. Further fully details are provided in Supplementary Table 11.

The results of the Network Meta-Analysis indicated that curcumin [MD = −5.02, 95% CI (−8.25, −1.80)] was superior to the placebo in reducing TJC. The probability ranking of various plant active substance interventions for decreasing TJC identified curcumin as the highest (SUCRA: 76.2%, as shown in Figure 7B). A comparison of the different interventions is provided in Table 6.

**TABLE 6:** Treatments ranked by SUCRA values and the league table of their relative effectiveness in terms of TJC.

Curcumin SUCRA 76.2%									
−0.63 (−8.87, 7.60)	TwRE SUCRA 66%								
−0.82 (−8.95, 7.31)	−0.19 (−11.75, 11.38)	Pomegranate extract SUCRA 64.4%							
−1.55 (−10.85, 7.74)	−0.92 (−8.89, 7.06)	−0.73 (−13.06, 11.60)	SIN SUCRA 56.8%						
−2.42 (−9.60, 4.75)	−1.79 (−12.70, 9.12)	−1.60 (−11.43, 8.23)	−0.87 (−12.59, 10.85)	Resveratrol SUCRA 52.4%					
−2.45 (−10.94, 6.03)	−1.82 (−13.64, 10.00)	−1.63 (−12.46, 9.20)	−0.90 (−13.47, 11.67)	−0.03 (−10.16, 10.10)	Sesamin SUCRA 51.2%				
−2.54 (−9.31, 4.22)	−1.91 (−6.61, 2.79)	−1.72 (−12.29, 8.85)	−0.99 (−7.44, 5.46)	−0.12 (−9.97, 9.73)	−0.09 (−10.93, 10.75)	Usual treatment SUCRA 47.6%			
−4.52 (−11.69, 2.65)	−3.89 (−14.80, 7.02)	−3.70 (−13.53, 6.13)	−2.97 (−14.69, 8.75)	−2.10 (−11.16, 6.96)	−2.07 (−12.20, 8.06)	−1.98 (−11.83, 7.87)	Quercetin SUCRA 33.9%		
−5.52 (−12.72, 1.68)	−4.89 (−15.82, 6.04)	−4.70 (−14.55, 5.16)	−3.97 (−15.71, 7.77)	−3.10 (−12.18, 5.98)	−3.07 (−13.22, 7.08)	−2.98 (−12.85, 6.89)	−1.00 (−10.08, 8.08)	TGP SUCRA 26.3%	
−5.02 (−8.25,-1.80)	−4.39 (−13.23, 4.45)	−4.20 (−11.66, 3.26)	−3.47 (−13.30, 6.35)	−2.60 (−9.01, 3.80)	−2.57 (−10.42, 5.28)	−2.48 (−9.97, 5.00)	−0.50 (−6.90, 5.90)	0.50 (−5.94, 6.93)	Placebo SUCRA 25.2%

Abbreviations: TwRE, *Tripterygium wilfordii* root extract; SIN, sinomenine; TGP, total glucosides of paeony.

### Publication bias test

**FIGURE 8:**
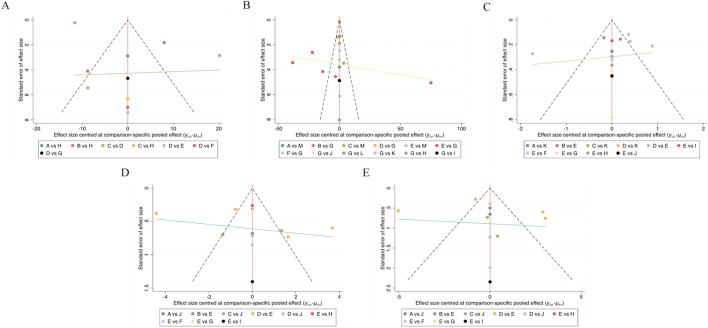
Funnel plot on publication bias. **(A)** VAS; **(B)** inflammatory marker; **(C)** disease activity; **(D)** SJC; **(E)** TJC.

The presence of publication bias was assessed by creating a funnel plot for each outcome; visual analysis revealed no evidence of this bias (Wallace et al., 2009). Further details are shown in Figure 8.

## Discussion

This network meta-analysis was performed to assess the efficacy of various plant active substances in treating RA. The main aim of this study was to evaluate the efficacy of different plant active substances in RA patients. This analysis included 18 studies, covering 10 distinct treatment modalities and involving 1,674 RA patients. The results indicated that curcumin showed significantly greater efficacy in reducing swollen joint count (SJC) and tender joint count (TJC) compared to placebo. Curcumin was also more effective in reducing inflammatory markers. Quercetin was more effective in reducing Visual Analog Scale (VAS) scores, while resveratrol was more effective in reducing the Disease Activity Score for 28 joints (DAS28).

Regarding VAS levels, our study found that quercetin exhibited superior efficacy. The VAS is employed to assess pain intensity in patients with RA (Dequeker and Wuestenraed, 1986). Historically, pain in RA has been associated with inflammatory processes in the joints. However, accumulating data suggests that neurochemical changes within the sensory nervous system and altered central pain processing are also likely involved (Bas et al., 2016). Quercetin, a leading phenolic compound and the most common bioflavonoid, is widely present in many fruits and vegetables (Cao et al., 2009). Quercetin has potent anti-inflammatory effects (Alam et al., 2014; McAnulty et al., 2013), suppressing the expression of inflammatory cytokines, notably TNF-α and IL-2 (Boots et al., 2008; Yu et al., 2008). Quercetin not only alleviates inflammatory pain but also provides a consistent analgesic effect on neuropathic pain. This effect may be attributed to its modulation of GABA_A, GABA_B, and 5-HT receptors, as well as the endogenous release of glucocorticoids (Filho et al., 2008; Martínez et al., 2009). Our findings suggest that quercetin may show increased efficacy in terms of reducing VAS scores. However, there is need for further research to validate these results.

In our research on inflammatory markers, as well as SJC and TJC, we found that curcumin was highly effective in improving these indicators in RA patients. ESR and CRP are systemic inflammatory markers used to determine the degree of RA activity (Anusha et al., 2019). SJC and TJC are among the simplest measurable clinical markers of disease severity and activity in RA (Inderjeeth et al., 2019). Curcumin, a naturally occurring compound extracted from the roots of turmeric, is one of the most bioactive constituents within the polyphenolic curcuminoid group (Benameur et al., 2021). It exerts antioxidant and anti-inflammatory effects without significant adverse effects (Hewlings and Kalman, 2017). *In vivo*, curcumin downregulated inflammatory cytokine levels (TNF-α and IL-17) and attenuated joint inflammation in collagen-induced arthritis (CIA) rats (Wang et al., 2019). *In vitro* and in preclinical models, curcumin modulates pain by affecting IL-10 secretion, a cytokine with strong anti-inflammatory and immunosuppressive effects, produced by cells from both the innate and adaptive immune systems. (Mollazadeh et al., 2019). Curcumin attenuated the severity of arthritis and reduced synovial hyperplasia by modulating the inc00052/miR-126-5p/PIAS2 pathway through the JAK2/STAT3 signaling mechanism, thereby providing protective effects against RA (Xiao et al., 2022). Besides, curcumin alleviates CIA-induced inflammation and synovial hyperplasia via the mTOR pathway (Dai et al., 2018). Recent studies have demonstrated that curcumin exhibits significant efficacy in inhibiting inflammatory responses and alleviating symptoms such as pain and swelling in clinical trials (Zeng et al., 2022). These findings corroborate the anti-arthritic properties of curcumin and underscore its potential as a therapeutic agent in treating RA. Our research findings indicate that, among various plant active substances, curcumin demonstrates superior efficacy in patients with RA. However, future research requires large-scale clinical trials to evaluate the effects of curcumin in RA patients.

Our study indicates that resveratrol may be more effective for RA patients, as evidenced by DAS28 scores. The DAS28 criterion, developed in 1995, comprises four components: the number of SJC, the number of TJC, the patient’s global health score, and ESR (Prevoo et al., 1995). DAS28 is the most widely used method for evaluating RA activity. Resveratrol, a natural antioxidant found in various plant species, has significant antioxidant and anti-inflammatory properties. Resveratrol suppresses oxidative stress in AA rats and increases mtROS production by reducing the autophagy proteins Beclin1 and the oxidative stress protein MnSOD, which promotes apoptosis in FLSs (Zhang et al., 2016). Resveratrol mitigated inflammation and cartilage degradation in the experimental IA model by inhibiting NF-κB activation and its associated gene expression. Furthermore, it suppressed the expression of COX-2 and iNOS by preventing the aberrant activation of NF-κB. Additionally, resveratrol broadly inhibited NF-κB activation induced by IL-1β and TNF-α (Holmes-McNary and Baldwin, 2000; Martinez and Moreno, 2000; Surh et al., 2001). However, due to the limited accuracy of DAS28 in evaluating RA, further high-quality research is necessary to validate our findings.

Overall, our research has clinical significance. Curcumin exhibited superior efficacy in reducing SJC and TJC compared to a placebo. It also showed greater effectiveness in reducing inflammatory markers. Quercetin exhibited greater effectiveness in reducing VAS. Resveratrol improved DAS28 scores, providing valuable insights for RA patients considering plant active substances. However, it is important to acknowledge that our conclusions are indicative rather than conclusive, and we cannot unreservedly recommend plant active substances for the treatment of RA. This highlights the necessity for subsequent studies with larger sample sizes to confirm and further extend our results.

### Strengths and limitations

Our study possesses several strengths: it encompasses 18 articles totaling 1,674 patients for a robust sample size. The study evaluates 10 plant active substances, employs stringent inclusion criteria for systematic literature retrieval, and adheres to established standards for systematic review and meta-analysis reporting.

Numerous studies have shown that plant active substances from herbal can improve RA disease activity, pain, and swelling without the side effects that routine treatment of RA with NSAIDs, biologics, and glucocorticoid (Sharma et al., 2021). When patients are faced with long-term treatments, the high cost of conventional therapies, and their serious side effects, plant active substances are a good option as an alternative treatment.

While our research offers valuable insights, it is constrained by several limitations. The efficacy of plant active substances on clinical outcomes is influenced by a multitude of variables, including baseline levels, stages of rheumatoid arthritis, treatment durations, dosages, body mass index (BMI), and other potential confounders. The inconsistency in reporting these factors complicates the interpretation of results. Furthermore, the wide range of dosages used for plant active substances across studies, combined with the absence of a standardized dosing protocol, may contribute to variability in outcomes. The impact of prior treatment regimens, such as DMARDs, also warrants consideration. Additionally, variations in extraction techniques and inadequate descriptions of these methodologies may introduce bias.

The conclusion posits that the hypothesis regarding the efficacy of plant active substances in treating RA is inadequately substantiated by rigorous clinical evidence, particularly due to the absence of direct comparative data for certain therapies. Consequently, the study’s findings should be interpreted with caution, highlighting the urgent need for more extensive and thorough research in this domain.

## Conclusion

The findings of the network meta-analysis (NMA) suggest potential therapeutic benefits of plant active substances, such as curcumin, quercetin, and resveratrol, for RA patients. This conclusion is substantiated by the comprehensive analysis of various treatment modalities and the subsequent determination of SUCRA scores. Specifically, curcumin exhibited superior efficacy in reducing SJC, TJC, and inflammatory markers, quercetin showed greater effectiveness in reducing VAS, while resveratrol was more effective in reducing DAS28.

## Data Availability

The original contributions presented in the study are included in the article/Supplementary Material, further inquiries can be directed to the corresponding authors.

## Abbreviations

RA, rheumatoid arthritis; VAS, Visual Analogue Scale; SJC, Swollen Joint Count; TJC, Tender Joint Count; DAS28, Disease Activity Score on 28 joints; SUCRA, Surface Under the Cumulative Ranking Curve; NMA, network meta-analysis; TwRE, *Tripterygium wilfordii* root extract; MTX, methotrexate; TGP, total glucosides of paeony; MKK3, mitogen kinase-3; p38α-MAPK, p38α mitogen-activated protein kinase; RUNX-2, runt-related transcription factor; RCTs, randomized controlled trials; PRISMA, Preferred Reporting Items for Systematic Reviews and Meta-Analyses; CRP, C-reactive protein; ESR, Erythrocyte Sedimentation Rate; ROB, risk of bias; SD, standard deviations; MD, mean difference; SMD, standardized mean difference; CI, confidence intervals; CIA, collagen-induced arthritis; BMI, body mass index.

## References

[B1] Alam M. M. Meerza D. Naseem I. (2014). Protective effect of quercetin on hyperglycemia, oxidative stress and DNA damage in alloxan induced type 2 diabetic mice. Life Sci. 109 (1), 8–14. 10.1016/j.lfs.2014.06.005 24946265

[B2] Amalraj A. Varma K. Jacob J. Divya C. Kunnumakkara A. B. Stohs S. J. (2017). A novel highly bioavailable curcumin formulation improves symptoms and diagnostic indicators in rheumatoid arthritis patients: a randomized, double-blind, placebo-controlled, two-dose, three-arm, and parallel-group study. J. Med. Food 20 (10), 1022–1030. 10.1089/jmf.2017.3930 28850308

[B3] Anusha D. Chaly P. E. Junaid M. Nijesh J. E. Shivashankar K. Sivasamy S. (2019). Efficacy of a mouthwash containing essential oils and curcumin as an adjunct to nonsurgical periodontal therapy among rheumatoid arthritis patients with chronic periodontitis: a randomized controlled trial. Indian J. Dent. Res. 30 (4), 506–511. 10.4103/ijdr.IJDR_662_17 31745043

[B4] Bas D. B. Su J. Wigerblad G. Svensson C. I. (2016). Pain in rheumatoid arthritis: models and mechanisms. Pain Manag. 6 (3), 265–284. 10.2217/pmt.16.4 27086843

[B5] Benameur T. Giacomucci G. Panaro M. A. Ruggiero M. Trotta T. Monda V. (2021). New promising therapeutic avenues of curcumin in brain diseases. Molecules 27 (1), 236. 10.3390/molecules27010236 35011468 PMC8746812

[B6] Bider C. M. Matt K. Irving M. Hook G. Yusen J. EaLyar F. (2007). Olive extract supplement decreases pain and improves daily activities in adults with osteoarthritis and decreases plasma homocysteine in those with rheumatoid arthritis. Nutr. Res. 27 (8), 470–477. 10.1016/j.nutres.2007.06.003

[B7] Boots A. W. Wilms L. C. Swennen E. L. Kleinjans J. C. Bast A. Haenen G. R. (2008). *In vitro* and *ex vivo* anti-inflammatory activity of quercetin in healthy volunteers. Nutrition 24 (7-8), 703–710. 10.1016/j.nut.2008.03.023 18549926

[B8] Burmester G. R. Pope J. E. (2017). Novel treatment strategies in rheumatoid arthritis. Lancet 389 (10086), 2338–2348. 10.1016/s0140-6736(17)31491-5 28612748

[B9] Cao H. Wu D. Wang H. Xu M. (2009). Effect of the glycosylation of flavonoids on interaction with protein. Spectrochim. Acta A Mol. Biomol. Spectrosc. 73 (5), 972–975. 10.1016/j.saa.2009.05.004 19493695

[B10] Chaimani A. Higgins J. P. Mavridis D. Spyridonos P. Salanti G. (2013). Graphical tools for network meta-analysis in STATA. PLoS One 8 (10), e76654. 10.1371/journal.pone.0076654 24098547 PMC3789683

[B11] Chandran B. Goel A. (2012). A randomized, pilot study to assess the efficacy and safety of curcumin in patients with active rheumatoid arthritis. Phytother. Res. 26 (11), 1719–1725. 10.1002/ptr.4639 22407780

[B12] Chatterjee A. Jayaprakasan M. Chakrabarty A. K. Lakkaniga N. R. Bhatt B. N. Banerjee D. (2024). Comprehensive insights into rheumatoid arthritis: pathophysiology, current therapies and herbal alternatives for effective disease management. Phytother. Res. 38 (6), 2764–2799. 10.1002/ptr.8187 38522945

[B13] Chen Z. Li X. P. Li Z. J. Xu L. Li X. M. (2012). Reduced hepatotoxicity by total glucosides of paeony in combination treatment with leflunomide and methotrexate for patients with active rheumatoid arthritis [Conference Abstract]. Int. J. Rheumatic Dis. 15, 69. 10.1111/j.1756-185X.2012.01785.x 23415907

[B14] Chen Z. Li X. P. Li Z. J. Xu L. Li X. M. (2013). Reduced hepatotoxicity by total glucosides of paeony in combination treatment with leflunomide and methotrexate for patients with active rheumatoid arthritis. Int. Immunopharmacol. 15 (3), 474–477. 10.1016/j.intimp.2013.01.021 23415907

[B15] Dai Q. Zhou D. Xu L. Song X. (2018). Curcumin alleviates rheumatoid arthritis-induced inflammation and synovial hyperplasia by targeting mTOR pathway in rats. Drug Des. Devel Ther. 12, 4095–4105. 10.2147/dddt.S175763 PMC6284537 30584274

[B16] Dequeker J. Wuestenraed L. (1986). The effect of biometeorological factors on Ritchie articular index and pain in rheumatoid arthritis. Scand. J. Rheumatol. 15 (3), 280–284. 10.3109/03009748609092593 3798044

[B17] Dong Y. Cao W. Cao J. (2021). Treatment of rheumatoid arthritis by phototherapy: advances and perspectives. Nanoscale 13 (35), 14591–14608. 10.1039/d1nr03623h 34473167

[B18] Filho A. W. Filho V. C. Olinger L. de Souza M. M. (2008). Quercetin: further investigation of its antinociceptive properties and mechanisms of action. Arch. Pharm. Res. 31 (6), 713–721. 10.1007/s12272-001-1217-2 18563352

[B19] Ghavipour M. Sotoudeh G. Tavakoli E. Mowla K. Hasanzadeh J. Mazloom Z. (2017). Pomegranate extract alleviates disease activity and some blood biomarkers of inflammation and oxidative stress in Rheumatoid Arthritis patients. Eur. J. Clin. Nutr. 71 (1), 92–96. 10.1038/ejcn.2016.151 27577177

[B20] Goldbach-Mansky R. Wilson M. Fleischmann R. Olsen N. Silverfield J. Kempf P. (2009). Comparison of *Tripterygium wilfordii* hook F versus sulfasalazine in the treatment of rheumatoid arthritis A randomized trial. Ann. Intern. Med. 151 (4), 229–240. 10.7326/0003-4819-151-4-200908180-00005 19687490 PMC2938780

[B21] Hang Y. Qin X. Ren T. Cao J. (2018). Baicalin reduces blood lipids and inflammation in patients with coronary artery disease and rheumatoid arthritis: a randomized, double-blind, placebo-controlled trial. Lipids Health Dis. 17 (1), 146. 10.1186/s12944-018-0797-2 29935544 PMC6015450

[B22] Helli B. Shahi M. M. Mowla K. Jalali M. T. Haghighian H. K. (2019). A randomized, triple-blind, placebo-controlled clinical trial, evaluating the sesamin supplement effects on proteolytic enzymes, inflammatory markers, and clinical indices in women with rheumatoid arthritis. Phytother. Res. 33 (9), 2421–2428. 10.1002/ptr.6433 31309643

[B23] Hemmati A. A. Rajaee E. Houshmand G. Fakhroddin M. A. Dargahi-MalAmir M. Hesam S. (2016). Study the effects of anti-inflammatory curcumex capsules containing three plants (ginger, curcumin and black pepper) in patients with active Rheumatoid Arthritis [Article]. IIOAB J. 7, 389–392. Available at: https://www.researchgate.net/publication/313768741.

[B24] Hewlings S. J. Kalman D. S. (2017). Curcumin: a review of its effects on human health. Foods 6 (10), 92. 10.3390/foods6100092 29065496 PMC5664031

[B25] Higgins J. P. Altman D. G. Gøtzsche P. C. Jüni P. Moher D. Oxman A. D. (2011). The Cochrane Collaboration's tool for assessing risk of bias in randomised trials. Bmj 343, d5928. 10.1136/bmj.d5928 22008217 PMC3196245

[B26] Holmes-McNary M. Baldwin A. S. Jr. (2000). Chemopreventive properties of trans-resveratrol are associated with inhibition of activation of the IkappaB kinase. Cancer Res. 60 (13), 3477–3483. 10.1046/j.1523-5394.2000.84005.x 10910059

[B27] Huang H. Qin J. Wen Z. Liu Y. Chen C. Wang C. (2024). Effects of natural extract interventions in prostate cancer: a systematic review and network meta-analysis. Phytomedicine 129, 155598. 10.1016/j.phymed.2024.155598 38608596

[B28] Inderjeeth C. A. Inderjeeth A. J. Raymond W. D. (2019). A multicentre observational study comparing patient reported outcomes to assess reliability of swollen and tender joint assessments and response to certolizumab treatment as compared to clinician assessments in rheumatoid arthritis. Int. J. Rheum. Dis. 22 (1), 73–80. 10.1111/1756-185x.13364 30187688

[B29] Jackson D. Riley R. White I. R. (2011). Multivariate meta-analysis: potential and promise. Stat. Med. 30 (20), 2481–2498. 10.1002/sim.4172 21268052 PMC3470931

[B30] Javadi F. Ahmadzadeh A. Eghtesadi S. Aryaeian N. Zabihiyeganeh M. Rahimi Foroushani A. (2017). The effect of quercetin on inflammatory factors and clinical symptoms in women with rheumatoid arthritis: a double-blind, randomized controlled trial. J. Am. Coll. Nutr. 36 (1), 9–15. 10.1080/07315724.2016.1140093 27710596

[B31] Khera R. Murad M. H. Chandar A. K. Dulai P. S. Wang Z. Prokop L. J. (2016). Association of pharmacological treatments for obesity with weight loss and adverse events: a systematic review and meta-analysis. JAMA 315 (22), 2424–2434. 10.1001/jama.2016.7602 27299618 PMC5617638

[B32] Khojah H. M. Ahmed S. Abdel-Rahman M. S. Elhakeim E. H. (2018). Resveratrol as an effective adjuvant therapy in the management of rheumatoid arthritis: a clinical study. Clin. Rheumatol. 37 (8), 2035–2042. 10.1007/s10067-018-4080-8 29611086

[B33] Li D. Chen P. (2021). Effects of aquatic exercise and land-based exercise on cardiorespiratory fitness, motor function, balance, and functional independence in stroke patients-A meta-analysis of randomized controlled trials. Brain Sci. 11 (8), 1097. 10.3390/brainsci11081097 34439716 PMC8394174

[B34] Lv Q. W. Zhang W. Shi Q. Zheng W. J. Li X. Chen H. (2015). Comparison of *Tripterygium wilfordii* Hook F with methotrexate in the treatment of active rheumatoid arthritis (TRIFRA): a randomised, controlled clinical trial. Ann. Rheumatic Dis. 74 (6), 1078–1086. 10.1136/annrheumdis-2013-204807 24733191

[B35] Marotta N. Demeco A. Moggio L. Marinaro C. Pino I. Barletta M. (2020). Comparative effectiveness of breathing exercises in patients with chronic obstructive pulmonary disease. Complement. Ther. Clin. Pract. 41, 101260. 10.1016/j.ctcp.2020.101260 33221632

[B36] Martínez A. L. González-Trujano M. E. Aguirre-Hernández E. Moreno J. Soto-Hernández M. López-Muñoz F. J. (2009). Antinociceptive activity of Tilia americana var. mexicana inflorescences and quercetin in the formalin test and in an arthritic pain model in rats. Neuropharmacology 56 (2), 564–571. 10.1016/j.neuropharm.2008.10.010 19027760

[B37] Martinez J. Moreno J. J. (2000). Effect of resveratrol, a natural polyphenolic compound, on reactive oxygen species and prostaglandin production. Biochem. Pharmacol. 59 (7), 865–870. 10.1016/s0006-2952(99)00380-9 10718345

[B38] McAnulty L. S. Miller L. E. Hosick P. A. Utter A. C. Quindry J. C. McAnulty S. R. (2013). Effect of resveratrol and quercetin supplementation on redox status and inflammation after exercise. Appl. Physiol. Nutr. Metab. 38 (7), 760–765. 10.1139/apnm-2012-0455 23980734

[B39] Moher D. Shamseer L. Clarke M. Ghersi D. Liberati A. Petticrew M. (2015). Preferred reporting items for systematic review and meta-analysis protocols (PRISMA-P) 2015 statement. Syst. Rev. 4 (1), 1. 10.1186/2046-4053-4-1 25554246 PMC4320440

[B40] Mollazadeh H. Cicero A. F. G. Blesso C. N. Pirro M. Majeed M. Sahebkar A. (2019). Immune modulation by curcumin: the role of interleukin-10. Crit. Rev. Food Sci. Nutr. 59 (1), 89–101. 10.1080/10408398.2017.1358139 28799796

[B41] Myasoedova E. Crowson C. S. Kremers H. M. Therneau T. M. Gabriel S. E. (2010). Is the incidence of rheumatoid arthritis rising? results from Olmsted County, Minnesota, 1955-2007. Arthritis Rheum. 62 (6), 1576–1582. 10.1002/art.27425 20191579 PMC2929692

[B42] Nikolakopoulou A. Higgins J. P. T. Papakonstantinou T. Chaimani A. Del Giovane C. Egger M. (2020). CINeMA: an approach for assessing confidence in the results of a network meta-analysis. PLoS Med. 17 (4), e1003082. 10.1371/journal.pmed.1003082 32243458 PMC7122720

[B43] Papakonstantinou T. Nikolakopoulou A. Higgins J. P. T. Egger M. Salanti G. (2020). CINeMA: software for semiautomated assessment of the confidence in the results of network meta-analysis. Campbell Syst. Rev. 16 (1), e1080. 10.1002/cl2.1080 37131978 PMC8356302

[B44] Pourhabibi-Zarandi F. Rafraf M. Zayeni H. Asghari-Jafarabadi M. Ebrahimi A. A. (2022). Effects of curcumin supplementation on metabolic parameters, inflammatory factors and obesity values in women with rheumatoid arthritis: a randomized, double-blind, placebo-controlled clinical trial. Phytother. Res. 36 (4), 1797–1806. 10.1002/ptr.7422 35178811

[B45] Pourhabibi-Zarandi F. Rafraf M. Zayeni H. Asghari-Jafarabadi M. Ebrahimi A. A. (2024). The efficacy of curcumin supplementation on serum total antioxidant capacity, malondialdehyde, and disease activity in women with rheumatoid arthritis: a randomized, double-blind, placebo-controlled clinical trial. Phytother. Res. 38, 3552–3563. 10.1002/ptr.8225 38699839

[B46] Prevoo M. L. van 't Hof M. A. Kuper H. H. van Leeuwen M. A. van de Putte L. B. van Riel P. L. (1995). Modified disease activity scores that include twenty-eight-joint counts. Development and validation in a prospective longitudinal study of patients with rheumatoid arthritis. Arthritis Rheum. 38 (1), 44–48. 10.1002/art.1780380107 7818570

[B47] Radner H. Smolen J. S. Aletaha D. (2011). Comorbidity affects all domains of physical function and quality of life in patients with rheumatoid arthritis. Rheumatol. Oxf. 50 (2), 381–388. 10.1093/rheumatology/keq334 21036875

[B48] Rasheed Z. Akhtar N. Haqqi T. M. (2010). Pomegranate extract inhibits the interleukin-1β-induced activation of MKK-3, p38α-MAPK and transcription factor RUNX-2 in human osteoarthritis chondrocytes. Arthritis Res. Ther. 12 (5), R195. 10.1186/ar3166 20955562 PMC2991031

[B49] Rezaieyazdi Z. Sarraf A. Khodashahi M. Sahebari M. Jarahi L. Rahimi H. R. (2023). Effect of oral curcumin on rheumatoid arthritis patients: a double-blind randomized clinical trial. J. Adv. Med. Biomed. Res. 31 (148), 432–440. [Article]. 10.30699/jambs.31.148.432

[B50] Rouse B. Chaimani A. Li T. (2017). Network meta-analysis: an introduction for clinicians. Intern Emerg. Med. 12 (1), 103–111. 10.1007/s11739-016-1583-7 27913917 PMC5247317

[B51] Safiri S. Kolahi A. A. Hoy D. Smith E. Bettampadi D. Mansournia M. A. (2019). Global, regional and national burden of rheumatoid arthritis 1990-2017: a systematic analysis of the Global Burden of Disease study 2017. Ann. Rheum. Dis. 78 (11), 1463–1471. 10.1136/annrheumdis-2019-215920 31511227

[B52] Salanti G. Ades A. E. Ioannidis J. P. (2011). Graphical methods and numerical summaries for presenting results from multiple-treatment meta-analysis: an overview and tutorial. J. Clin. Epidemiol. 64 (2), 163–171. 10.1016/j.jclinepi.2010.03.016 20688472

[B53] Sharma D. Chaubey P. Suvarna V. (2021). Role of natural products in alleviation of rheumatoid arthritis-A review. J. Food Biochem. 45 (4), e13673. 10.1111/jfbc.13673 33624882

[B54] Shi Y. Pan H. D. Wu J. L. Zou Q. H. Xie X. Y. Li H. G. (2022). The correlation between decreased ornithine level and alleviation of rheumatoid arthritis patients assessed by a randomized, placebo-controlled, double-blind clinical trial of sinomenine. Engineering 16, 93–99. 10.1016/j.eng.2021.04.014

[B55] Smolen J. S. Aletaha D. McInnes I. B. (2016). Rheumatoid arthritis. Lancet 388 (10055), 2023–2038. 10.1016/s0140-6736(16)30173-8 27156434

[B56] Surh Y. J. Chun K. S. Cha H. H. Han S. S. Keum Y. S. Park K. K. (2001). Molecular mechanisms underlying chemopreventive activities of anti-inflammatory phytochemicals: down-regulation of COX-2 and iNOS through suppression of NF-kappa B activation. Mutat. Res. 480-481, 243–268. 10.1016/s0027-5107(01)00183-x 11506818

[B57] Tian J. Chen J. W. Gao J. S. Li L. Xie X. (2013). Resveratrol inhibits TNF-α-induced IL-1β, MMP-3 production in human rheumatoid arthritis fibroblast-like synoviocytes via modulation of PI3kinase/Akt pathway. Rheumatol. Int. 33 (7), 1829–1835. 10.1007/s00296-012-2657-0 23328930

[B58] Tong B. Yu J. Wang T. Dou Y. Wu X. Kong L. (2015). Sinomenine suppresses collagen-induced arthritis by reciprocal modulation of regulatory T cells and Th17 cells in gut-associated lymphoid tissues. Mol. Immunol. 65 (1), 94–103. 10.1016/j.molimm.2015.01.014 25656802

[B59] Vats D. Flegal J. M. Jones G. L. (2019). Multivariate output analysis for Markov chain Monte Carlo. Oxford: Oxford Academic.

[B60] Wallace B. C. Schmid C. H. Lau J. Trikalinos T. A. (2009). Meta-Analyst: software for meta-analysis of binary, continuous and diagnostic data. BMC Med. Res. Methodol. 9, 80. 10.1186/1471-2288-9-80 19961608 PMC2795760

[B61] Wang Q. Ye C. Sun S. Li R. Shi X. Wang S. (2019). Curcumin attenuates collagen-induced rat arthritis via anti-inflammatory and apoptotic effects. Int. Immunopharmacol. 72, 292–300. 10.1016/j.intimp.2019.04.027 31005039

[B62] Xiang N. Li X. M. Zhang M. J. Zhao D. B. Zhu P. Zuo X. X. (2015). Total glucosides of paeony can reduce the hepatotoxicity caused by Methotrexate and Leflunomide combination treatment of active rheumatoid arthritis. Int. Immunopharmacol. 28 (1), 802–807. 10.1016/j.intimp.2015.08.008 26292180

[B63] Xiao J. Cai X. Zhou W. Wang R. Ye Z. (2022). Curcumin relieved the rheumatoid arthritis progression via modulating the linc00052/miR-126-5p/PIAS2 axis. Bioengineered 13 (4), 10973–10983. 10.1080/21655979.2022.2066760 35473503 PMC9208441

[B64] Yang M. Luo Y. Liu T. Zhong X. Yan J. Huang Q. (2018). The effect of puerarin on carotid intima-media thickness in patients with active rheumatoid arthritis: ARandomized controlled trial. Clin. Ther. 40 (10), 1752–1764. 10.1016/j.clinthera.2018.08.014 30245282

[B65] Yu E. S. Min H. J. An S. Y. Won H. Y. Hong J. H. Hwang E. S. (2008). Regulatory mechanisms of IL-2 and IFNgamma suppression by quercetin in T helper cells. Biochem. Pharmacol. 76 (1), 70–78. 10.1016/j.bcp.2008.03.020 18468581

[B66] Zeng L. Yang T. Yang K. Yu G. Li J. Xiang W. (2022). Curcumin and curcuma longa extract in the treatment of 10 types of autoimmune diseases: a systematic review and meta-analysis of 31 randomized controlled trials. Front. Immunol. 13, 896476. 10.3389/fimmu.2022.896476 35979355 PMC9376628

[B67] Zhang J. Song X. Cao W. Lu J. Wang X. Wang G. (2016). Autophagy and mitochondrial dysfunction in adjuvant-arthritis rats treatment with resveratrol. Sci. Rep. 6, 32928. 10.1038/srep32928 27611176 PMC5017199

