# Supplementary material for: Effects of plant active substances in rheumatoid arthritis—a systematic review and network meta-analysis
Source: Front Pharmacol. 2025 Feb 5;16:1536023. doi: 10.3389/fphar.2025.1536023 (PMC11835909; doi:10.3389/fphar.2025.1536023)
Supplement: Supplementary file 1 [file DataSheet1.docx]

**Table 1. The details of search strategy**

| Search | PubMed |
| --- | --- |
| #1 | Arthritis, Rheumatoid[MeSH Major Topic] |
| #2 | (Arthritis, Rheumatoid) OR (Rheumatoid arthritis) |
| #3 | (#1) OR (#2) |
| #4 | (((((((((((((((((((celastrol[MeSH Major Topic]) OR (Triptolide[MeSH Major Topic])) OR (Curcumin[MeSH Major Topic])) OR (Sinomenine[MeSH Major Topic])) OR (Piperlongumine[MeSH Major Topic])) OR (Baicalin[MeSH Major Topic])) OR (Tangeretin[MeSH Major Topic])) OR (Hesperetin[MeSH Major Topic])) OR (Matrine[MeSH Major Topic])) OR (Resveratrol[MeSH Major Topic])) OR (Genistein[MeSH Major Topic])) OR (Tanshinone[MeSH Major Topic])) OR (Dioscin[MeSH Major Topic])) OR (Silymarin[MeSH Major Topic])) OR (Berberine[MeSH Major Topic])) OR (Ginger extract[MeSH Major Topic])) OR (Hesperidin[MeSH Major Topic])) OR (Puerarin[MeSH Major Topic])) OR (Quercetin[MeSH Major Topic])) OR (Sesamin[MeSH Major Topic]) |
| #5 | (((((((((((((((((((((((((((((((((((((((((((((((((((((((((((((((((((((((((((Total glucosides of paeony) OR (celastrol)) OR (3-hydroxy-24-nor-2-oxo-1(10),3,5,7-friedelatetraen-29-oic acid)) OR (tripterin)) OR (tripterine)) OR (Tripterygium glycosides)) OR (Triptolide)) OR (Curcumin)) OR (Curcumin Phytosome)) OR (Phytosome, Curcumin)) OR (1,6-Heptadiene-3,5-dione, 1,7-bis(4-hydroxy-3-methoxyphenyl)-, (E,E)-)) OR (Diferuloylmethane)) OR (Turmeric Yellow)) OR (Yellow, Turmeric)) OR (Mervia)) OR (Sinomenine)) OR (Piperlongumine)) OR (Baicalin)) OR (Tangeretin)) OR (Hesperetin)) OR (Matrine)) OR (Matrine-type Alkaloids)) OR (Matrine type Alkaloids)) OR (Matrine Alkaloids)) OR (Matrine)) OR (Sophoridine)) OR (Matrine, (5beta)-Isomer)) OR (Resveratrol)) OR (3,4',5-Stilbenetriol)) OR (3,5,4'-Trihydroxystilbene)) OR (3,4',5-Trihydroxystilbene)) OR (trans-Resveratrol)) OR (trans Resveratrol)) OR (Resveratrol-3-sulfate)) OR (Resveratrol 3 sulfate)) OR (SRT 501)) OR (SRT-501)) OR (SRT501)) OR (cis-Resveratrol)) OR (cis Resveratrol)) OR (Resveratrol, (Z)-)) OR (trans-Resveratrol-3-O-sulfate)) OR (trans Resveratrol 3 O sulfate)) OR (Genistein)) OR (Genestein)) OR (Tanshinone)) OR (Dioscin)) OR (Silymarin)) OR (Silimarin)) OR (Carsil)) OR (Karsil)) OR (Legalon)) OR (Berberine)) OR (Umbellatine)) OR (pomegranate extract)) OR (Cinnamon extract)) OR (Cranberry extract)) OR (Crocus sativus L. extract)) OR (Garlic extract)) OR (Ginger extract)) OR (Hesperidin)) OR (Hesperidin 2S)) OR (2S, Hesperidin)) OR (4H-1-Benzopyran-4-one, 7-((6-O-(6-deoxy-alpha-L-mannopyranosyl)-beta-D-glucopyranosyl)oxy)-2,3-dihydro-5-hydroxy-2-(3-hydroxy-4-methoxyphenyl)-, (S)-)) OR (Hesperetin-7-Rutinoside)) OR (Hesperetin 7 Rutinoside)) OR (Hesperetin 7-Rhamnoglucoside)) OR (7-Rhamnoglucoside, Hesperetin)) OR (Hesperetin 7 Rhamnoglucoside)) OR (Puerarin)) OR (Quercetin)) OR (3,3',4',5,7-Pentahydroxyflavone)) OR (Dikvertin)) OR (Sesamin)) OR (Tea polyphenols)) OR (resin extract of Boswellia serrata) |
| #6 | (#4) OR (#5) |
| #7 | (#3) AND (#6) |

| Search | Embase |
| --- | --- |
| #1 | 'arthritis, rheumatoid':ab,ti OR 'rheumatoid arthritis':ab,ti |
| #2 | 'total glucosides of paeony':ab,ti OR celastrol:ab,ti OR tripterin:ab,ti OR tripterine:ab,ti OR 'tripterygium glycosides':ab,ti OR triptolide:ab,ti OR curcumin:ab,ti OR 'curcumin phytosome':ab,ti OR 'phytosome, curcumin':ab,ti OR diferuloylmethane:ab,ti OR 'turmeric yellow':ab,ti OR 'yellow, turmeric':ab,ti OR mervia:ab,ti OR sinomenine:ab,ti OR piperlongumine:ab,ti OR baicalin:ab,ti OR tangeretin:ab,ti OR hesperetin:ab,ti OR 'matrine-type alkaloids':ab,ti OR 'matrine type alkaloids':ab,ti OR 'matrine alkaloids':ab,ti OR matrine:ab,ti OR sophoridine:ab,ti OR resveratrol:ab,ti OR 'trans resveratrol':ab,ti OR 'resveratrol 3 sulfate':ab,ti OR 'srt 501':ab,ti OR srt501:ab,ti OR 'cis resveratrol':ab,ti OR 'trans resveratrol 3 o sulfate':ab,ti OR genistein:ab,ti OR genestein:ab,ti OR tanshinone:ab,ti OR dioscin:ab,ti OR silymarin:ab,ti OR silimarin:ab,ti OR carsil:ab,ti OR karsil:ab,ti OR legalon:ab,ti OR berberine:ab,ti OR umbellatine:ab,ti OR 'pomegranate extract':ab,ti OR 'cinnamon extract':ab,ti OR 'cranberry extract':ab,ti OR 'crocus sativus l. extract':ab,ti OR 'garlic extract':ab,ti OR 'ginger extract':ab,ti OR hesperidin:ab,ti OR 'hesperidin 2s':ab,ti OR '2s, hesperidin':ab,ti OR 'hesperetin 7 rutinoside':ab,ti OR 'hesperetin 7-rhamnoglucoside':ab,ti OR '7-rhamnoglucoside, hesperetin':ab,ti OR 'hesperetin 7 rhamnoglucoside':ab,ti OR puerarin:ab,ti OR quercetin:ab,ti OR dikvertin:ab,ti OR sesamin:ab,ti OR 'tea polyphenols':ab,ti OR 'resin extract of boswellia serrata':ab,ti |
| #3 | #1 AND #2 |

| Search | The Cochrane Library |
| --- | --- |
| #1 | (Arthritis, Rheumatoid):ti,ab,kw OR (Rheumatoid arthritis):ti,ab,kw |
| #2 | (Total glucosides of paeony):ti,ab,kw OR (celastrol):ti,ab,kw OR (tripterin):ti,ab,kw OR (tripterine):ti,ab,kw OR (Tripterygium glycosides):ti,ab,kw |
| #3 | (Triptolide):ti,ab,kw OR (Curcumin):ti,ab,kw OR (Curcumin Phytosome):ti,ab,kw OR (Phytosome, Curcumin):ti,ab,kw OR (Diferuloylmethane):ti,ab,kw |
| #4 | (Turmeric Yellow):ti,ab,kw OR (Yellow, Turmeric):ti,ab,kw OR (Mervia):ti,ab,kw OR (Sinomenine):ti,ab,kw OR (Piperlongumine):ti,ab,kw |
| #5 | (Baicalin):ti,ab,kw OR (Tangeretin):ti,ab,kw OR (Hesperetin):ti,ab,kw OR (Matrine):ti,ab,kw OR (Matrine-type Alkaloids):ti,ab,kw |
| #6 | (Matrine type Alkaloids):ti,ab,kw OR (Matrine Alkaloids):ti,ab,kw OR (Matrine):ti,ab,kw OR (Sophoridine):ti,ab,kw OR (Resveratrol):ti,ab,kw |
| #7 | (trans Resveratrol 3 O sulfate):ti,ab,kw OR (Genistein):ti,ab,kw OR (Genestein):ti,ab,kw OR (Tanshinone):ti,ab,kw OR (Dioscin):ti,ab,kw |
| #8 | (Silymarin):ti,ab,kw OR (Silimarin):ti,ab,kw OR (Carsil):ti,ab,kw OR (Karsil):ti,ab,kw OR (Legalon):ti,ab,kw |
| #9 | (Berberine):ti,ab,kw OR (Umbellatine):ti,ab,kw OR (pomegranate extract):ti,ab,kw OR (Cinnamon extract):ti,ab,kw OR (Cranberry extract):ti,ab,kw |
| #10 | (Crocus sativus L. extract):ti,ab,kw OR (Garlic extract):ti,ab,kw OR (Ginger extract):ti,ab,kw OR (Hesperidin):ti,ab,kw OR (Hesperidin 2S):ti,ab,kw |
| #11 | (Hesperetin 7 Rhamnoglucoside):ti,ab,kw OR (Puerarin):ti,ab,kw OR (Quercetin):ti,ab,kw OR (Dikvertin):ti,ab,kw OR (Sesamin):ti,ab,kw |
| #12 | (Tea polyphenols):ti,ab,kw OR (resin extract of Boswellia serrata):ti,ab,kw |
| #13 | #2 or #3 or #4 or #5 or #6 or #7 or #8 or #9 or #10 or #11 or #12 |
| #14 | #1 and #13 |


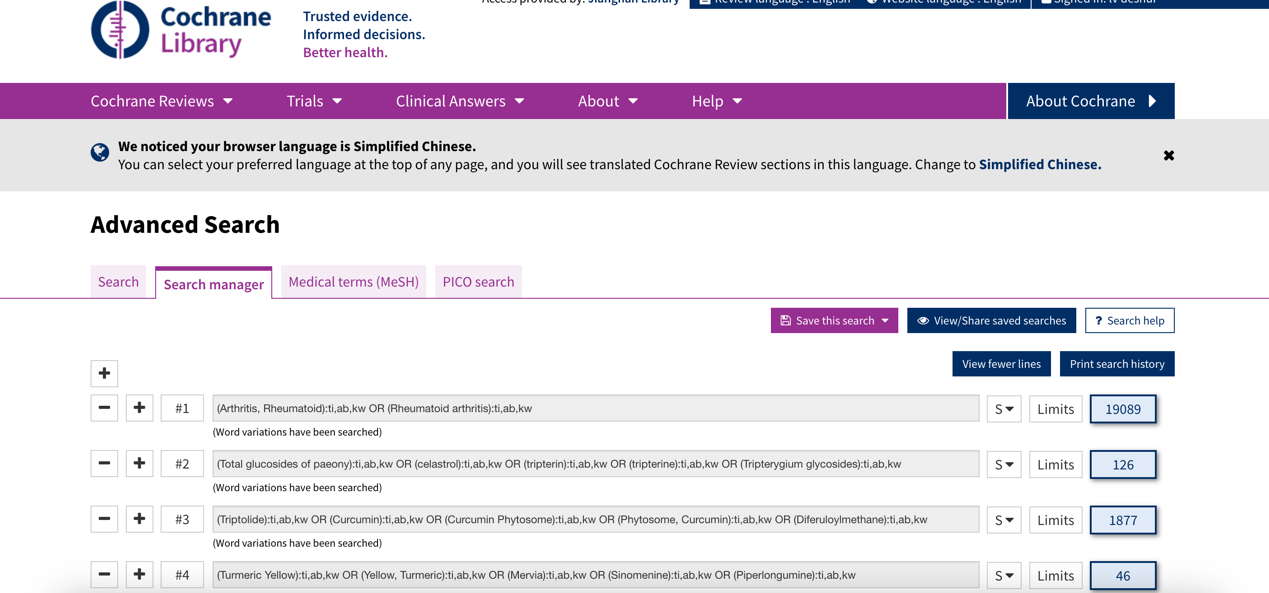


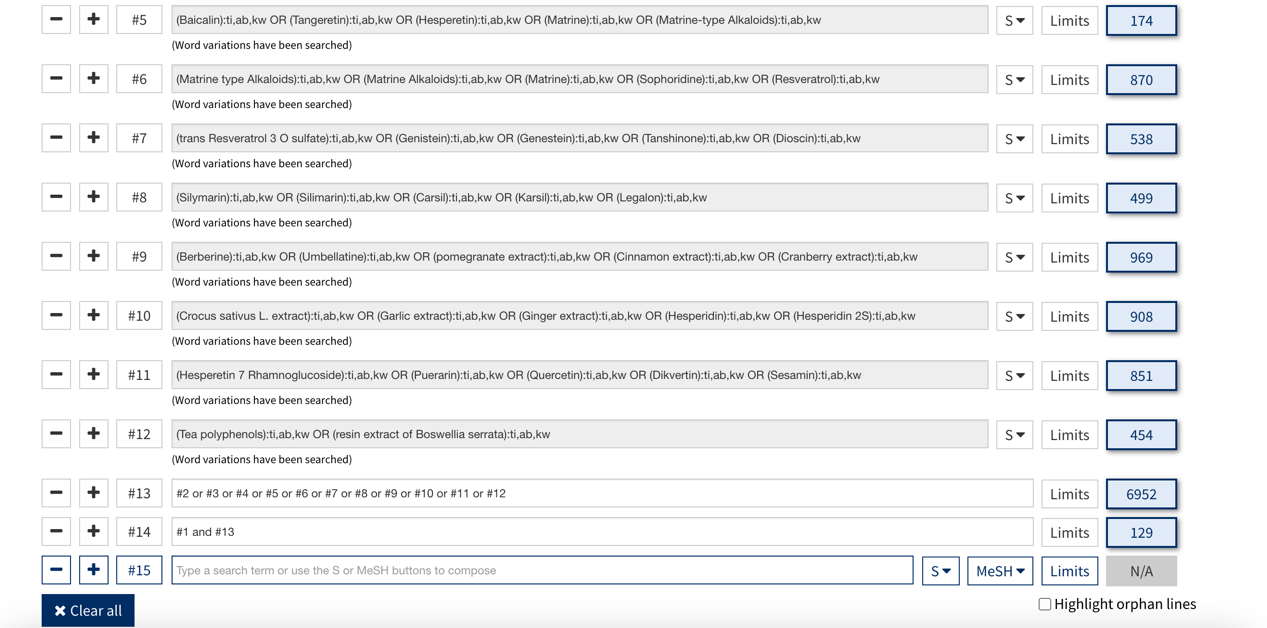


| Search | Web of Science |
| --- | --- |
| #1 | (ALL=(Arthritis, Rheumatoid)) OR ALL=(Rheumatoid arthritis) |
| #2 | (((((((((((((((((((((((((((((((((((((((((((((((((ALL=(Total glucosides of paeony)) OR ALL=(celastrol)) OR ALL=(tripterin)) OR ALL=(tripterine)) OR ALL=(Tripterygium glycosides)) OR ALL=(Triptolide)) OR ALL=(Curcumin)) OR ALL=(Curcumin Phytosome)) OR ALL=(Diferuloylmethane)) OR ALL=(Turmeric Yellow)) OR ALL=(Mervia)) OR ALL=(Sinomenine)) OR ALL=(Piperlongumine)) OR ALL=(Baicalin)) OR ALL=(Tangeretin)) OR ALL=(Hesperetin)) OR ALL=(Matrine)) OR ALL=(Matrine-type Alkaloids)) OR ALL=(Sophoridine)) OR ALL=(Resveratrol)) OR ALL=(trans-Resveratrol)) OR ALL=(Resveratrol-3-sulfate)) OR ALL=(SRT 501)) OR ALL=(cis-Resveratrol)) OR ALL=(trans-Resveratrol-3-O-sulfate)) OR ALL=(Genistein)) OR ALL=(Genestein)) OR ALL=(Tanshinone)) OR ALL=(Dioscin)) OR ALL=(Silymarin)) OR ALL=(Carsil)) OR ALL=(Karsil)) OR ALL=(Legalon)) OR ALL=(Berberine)) OR ALL=(Umbellatine)) OR ALL=(pomegranate extract)) OR ALL=(Cinnamon extract)) OR ALL=(Cranberry extract)) OR ALL=(Crocus sativus L. extract)) OR ALL=(Garlic extract)) OR ALL=(Ginger extract)) OR ALL=(Hesperidin)) OR ALL=(Hesperidin 2S)) OR ALL=(Hesperetin-7-Rutinoside)) OR ALL=(Puerarin)) OR ALL=(Quercetin)) OR ALL=(Dikvertin)) OR ALL=(Sesamin)) OR ALL=(Tea polyphenols)) OR ALL=(resin extract of Boswellia serrata) |
| #2 | #1 AND #2 |

| Search | Ovid |
| --- | --- |
| #1 | (ALL=(Arthritis, Rheumatoid)) OR ALL=(Rheumatoid arthritis) |


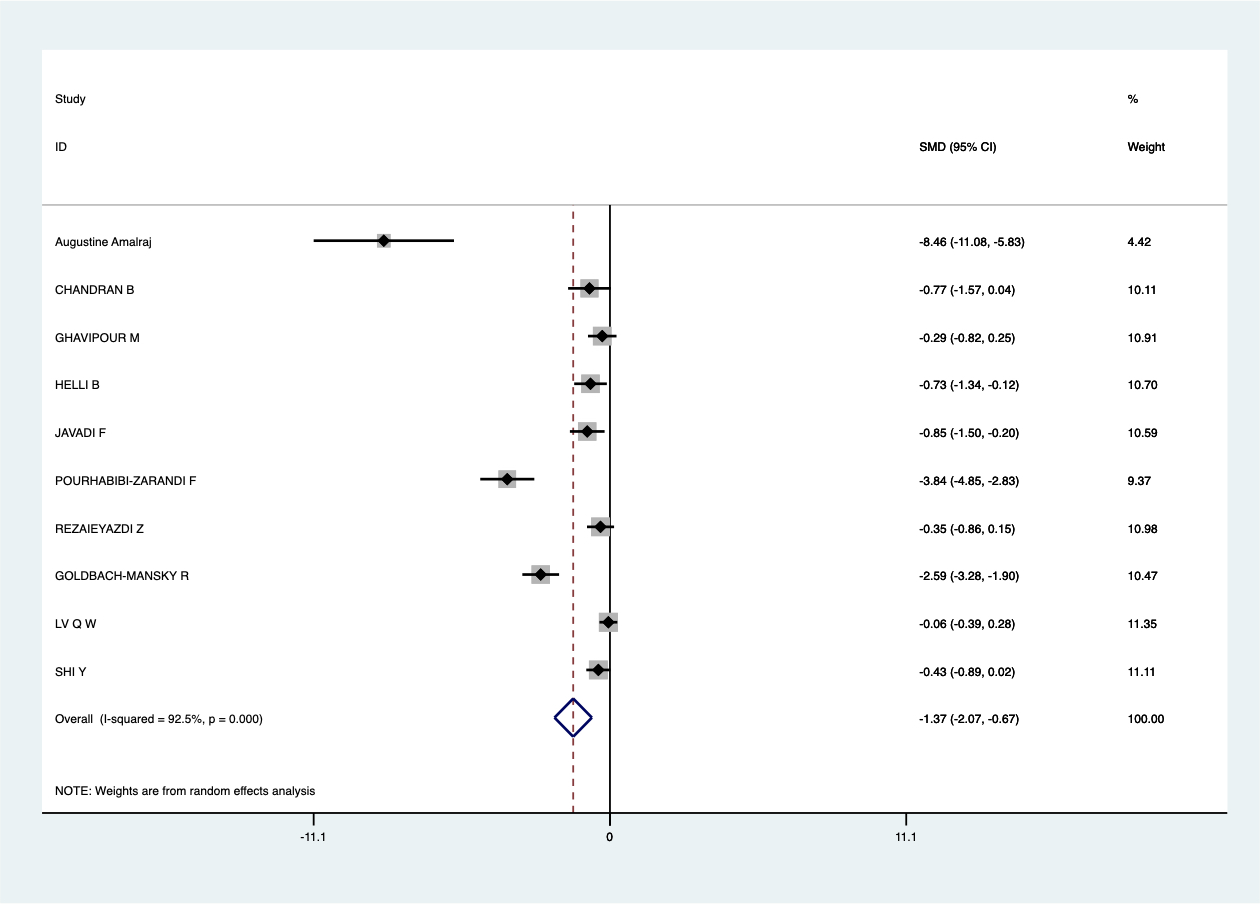


**Figure 1. Forest of VAS**


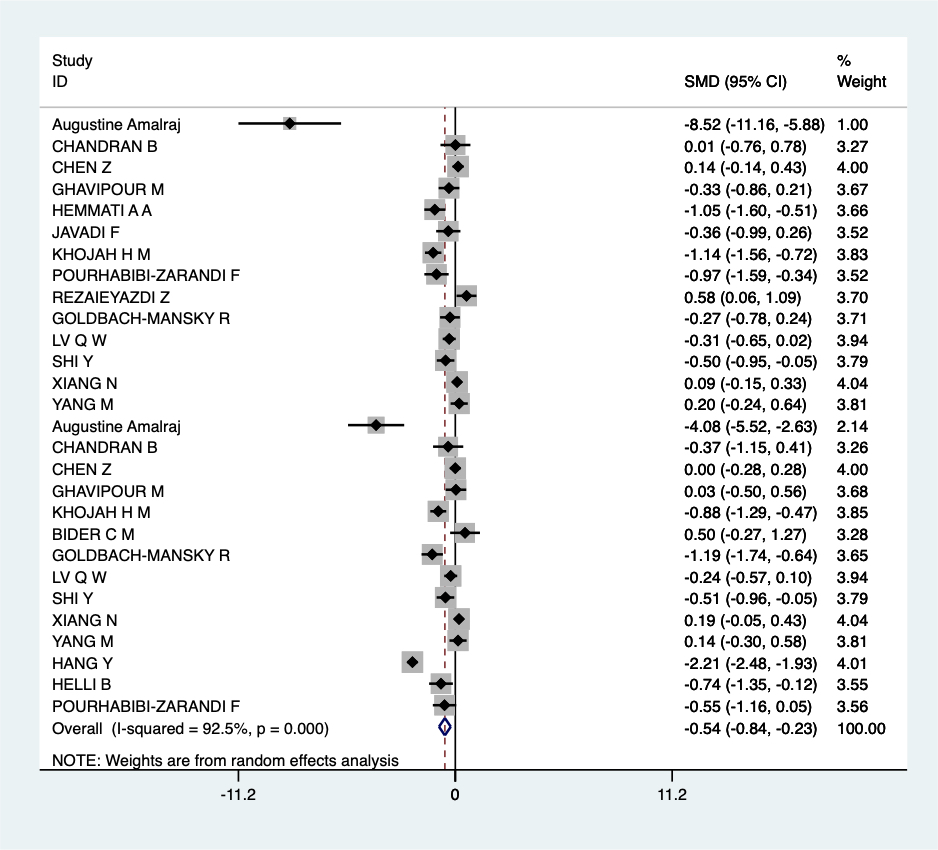


**Figure 2. Forest of inflammatory markers**


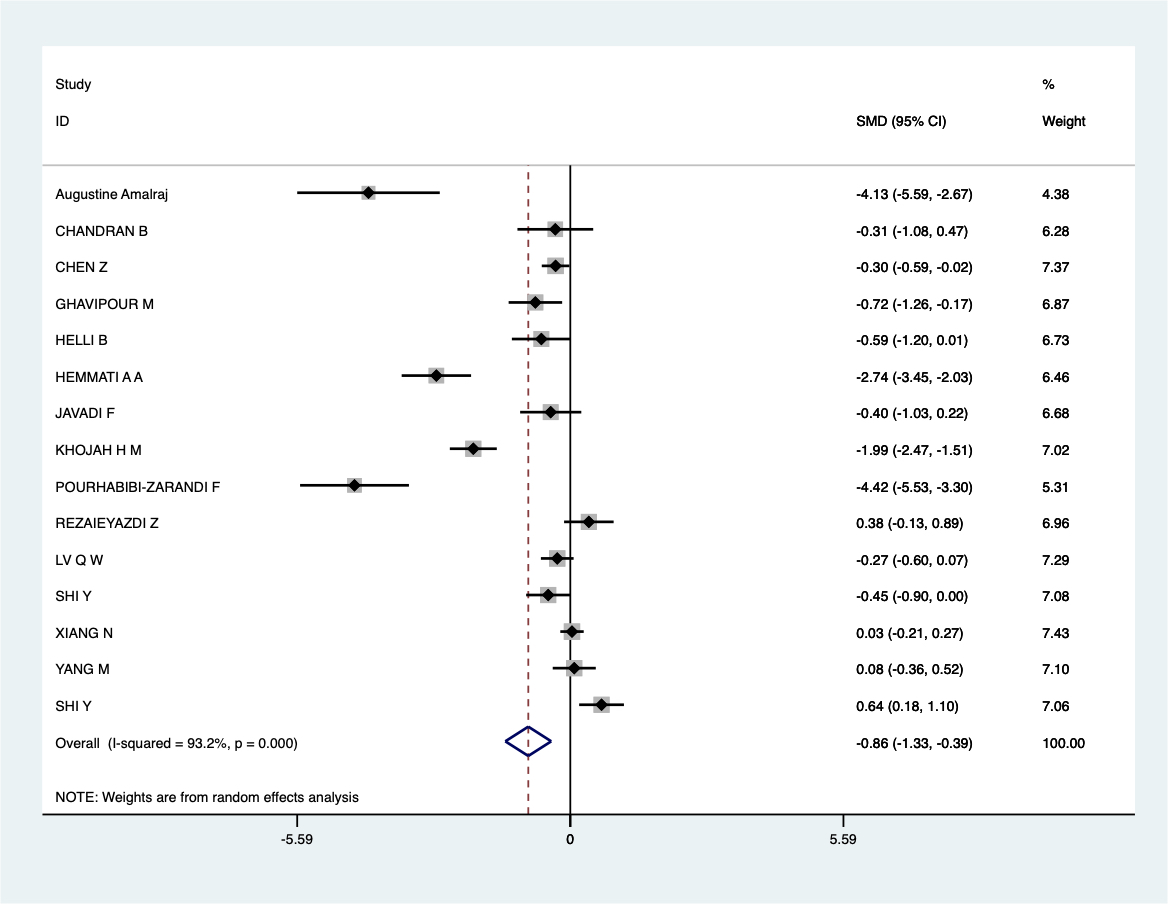


**Figure 3. Forest of DAS28**


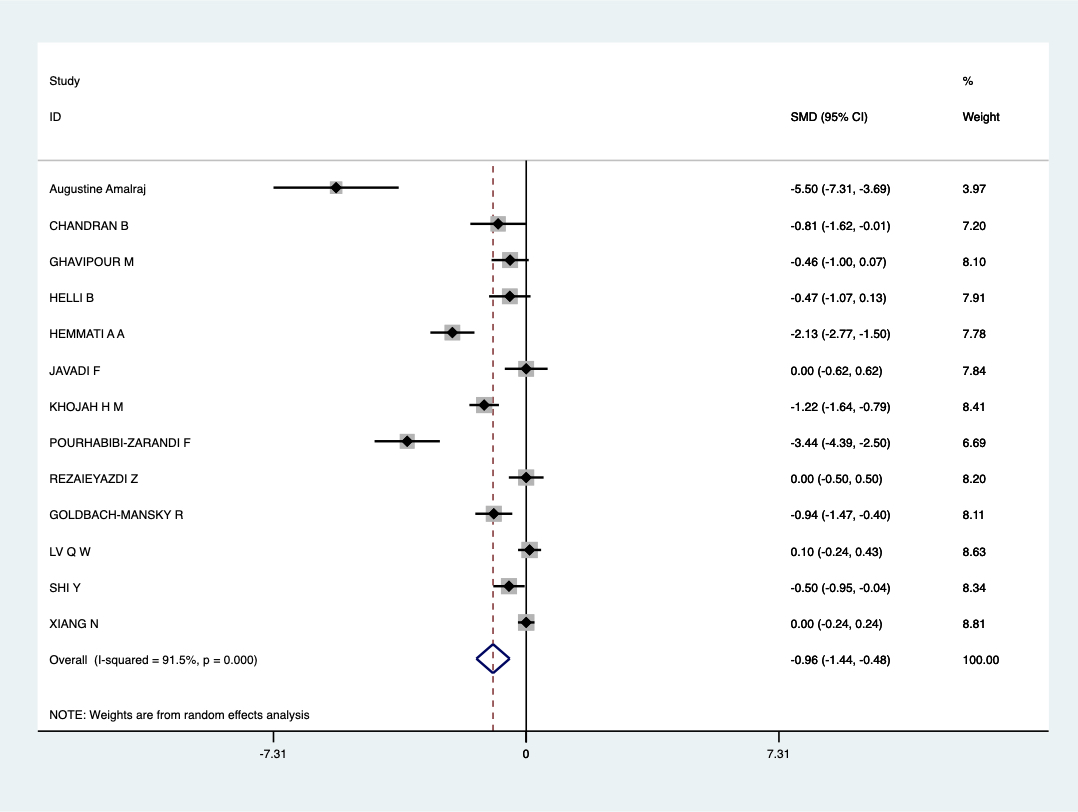

**Figure 4. Forest of SJC**


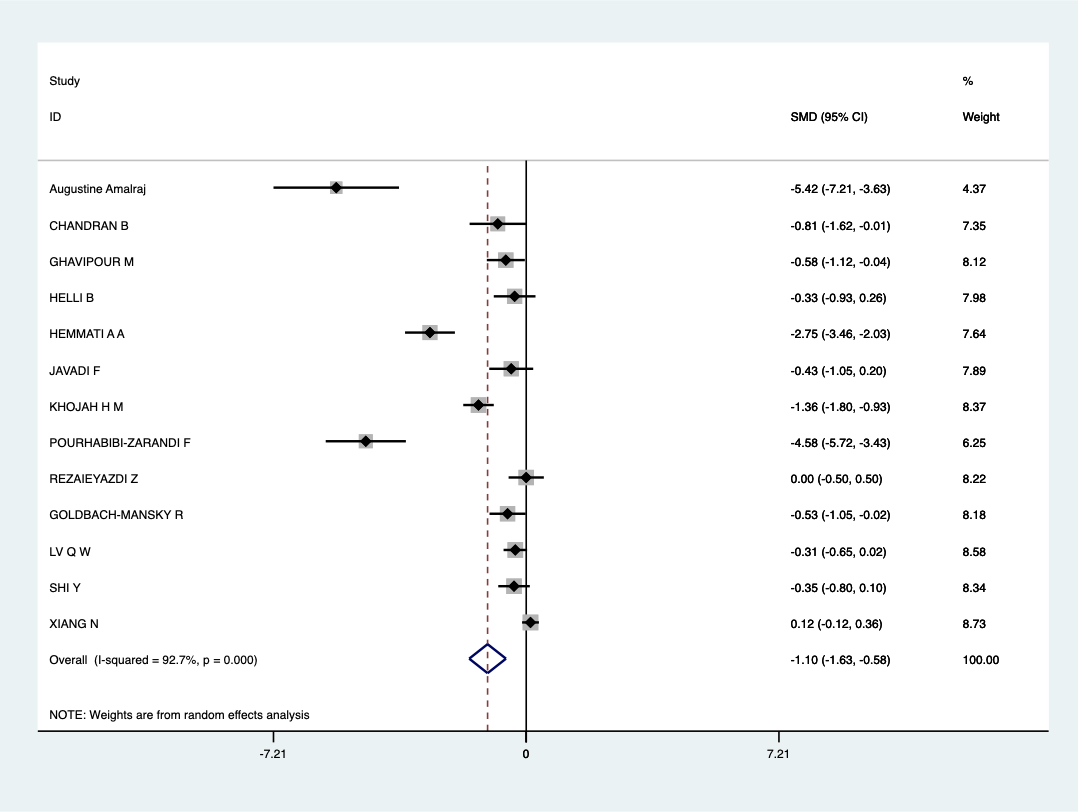


**Figure 5. Forest of TJC**

**Table 2. CINeMA Results of VAS**

| Comparison | Within-study bias | Reporting bias | Indirectness | Imprecision | Heterogeneity | Incoherence | Confidence rating |
| --- | --- | --- | --- | --- | --- | --- | --- |
| SIN:usual treatment | No concerns | Low risk | No concerns | Major concerns | No concerns | Major concerns | High |
| TwRE:usual treatment | Some concerns | Low risk | No concerns | No concerns | Major concerns | Major concerns | High |
| curcumin:placebo | No concerns | Low risk | No concerns | No concerns | Major concerns | Major concerns | High |
| curcumin:usual treatment | Major concerns | Low risk | Some concerns | Major concerns | No concerns | Major concerns | High |
| placebo:pomegranate extract | No concerns | Low risk | No concerns | Major concerns | No concerns | Major concerns | High |
| placebo:quercetin | Major concerns | Low risk | Some concerns | No concerns | Major concerns | Major concerns | High |
| placebo:sesamin | No concerns | Low risk | No concerns | No concerns | Major concerns | Major concerns | High |
| SIN:TwRE | No concerns | Low risk | No concerns | No concerns | Major concerns | Major concerns | High |
| curcumin:SIN | Some concerns | Low risk | Some concerns | Major concerns | No concerns | Major concerns | High |
| placebo:SIN | Some concerns | Low risk | No concerns | Major concerns | No concerns | Major concerns | High |
| pomegranate extract:SIN | No concerns | Low risk | No concerns | Major concerns | No concerns | Major concerns | High |
| quercetin:SIN | Some concerns | Low risk | No concerns | No concerns | Major concerns | Major concerns | High |
| sesamin:SIN | No concerns | Low risk | No concerns | Major concerns | No concerns | Major concerns | High |
| curcumin:TwRE | Some concerns | Low risk | Some concerns | Major concerns | No concerns | Major concerns | High |
| placebo:TwRE | Some concerns | Low risk | No concerns | Major concerns | No concerns | Major concerns | High |
| pomegranate extract:TwRE | Some concerns | Low risk | No concerns | Major concerns | No concerns | Major concerns | High |
| quercetin:TwRE | Some concerns | Low risk | No concerns | Major concerns | No concerns | Major concerns | High |
| sesamin:TwRE | Some concerns | Low risk | No concerns | Major concerns | No concerns | Major concerns | High |
| curcumin:pomegranate extract | No concerns | Low risk | No concerns | Major concerns | No concerns | Major concerns | High |
| curcumin:quercetin | Some concerns | Low risk | Some concerns | No concerns | Major concerns | Major concerns | High |
| curcumin:sesamin | No concerns | Low risk | No concerns | Major concerns | No concerns | Major concerns | High |
| placebo:usual treatment | Some concerns | Low risk | Some concerns | Major concerns | No concerns | Major concerns | High |
| pomegranate extract:quercetin | Some concerns | Low risk | Some concerns | Major concerns | No concerns | Major concerns | High |
| pomegranate extract:sesamin | No concerns | Low risk | No concerns | Major concerns | No concerns | Major concerns | High |
| pomegranate extract:usual treatment | Some concerns | Low risk | No concerns | Major concerns | No concerns | Major concerns | High |
| quercetin:sesamin | Some concerns | Low risk | Some concerns | Major concerns | No concerns | Major concerns | High |
| quercetin:usual treatment | Some concerns | Low risk | Some concerns | No concerns | Major concerns | Major concerns | High |
| sesamin:usual treatment | Some concerns | Low risk | No concerns | No concerns | Major concerns | Major concerns | High |

**Table 3. CINeMA Results of inflammatory markers**

| Comparison | Within-study bias | Reporting bias | Indirectness | Imprecision | Heterogeneity | Incoherence | Confidence rating |
| --- | --- | --- | --- | --- | --- | --- | --- |
| SIN:usual treatment | No concerns | Low risk | No concerns | Major concerns | No concerns | Major concerns | High |
| placebo:TGP | Some concerns | Low risk | No concerns | Major concerns | No concerns | Major concerns | High |
| TwRE:usual treatment | Some concerns | Low risk | No concerns | Major concerns | No concerns | Major concerns | High |
| baicalin:placebo | No concerns | Low risk | No concerns | Major concerns | No concerns | Major concerns | High |
| curcumin:placebo | No concerns | Low risk | No concerns | No concerns | Major concerns | Major concerns | High |
| curcumin:usual treatment | Major concerns | Low risk | Some concerns | Major concerns | No concerns | Major concerns | High |
| olive extract:placebo | Major concerns | Low risk | No concerns | Major concerns | No concerns | Major concerns | High |
| placebo:pomegranate extract | No concerns | Low risk | No concerns | Major concerns | No concerns | Major concerns | High |
| placebo:puerarin | Some concerns | Low risk | No concerns | Major concerns | No concerns | Major concerns | High |
| placebo:quercetin | Major concerns | Low risk | No concerns | Major concerns | No concerns | Major concerns | High |
| placebo:resveratrol | Major concerns | Low risk | No concerns | Major concerns | No concerns | Major concerns | High |
| placebo:sesamin | No concerns | Low risk | Some concerns | Major concerns | No concerns | Major concerns | High |
| SIN:TGP | Some concerns | Low risk | No concerns | Major concerns | No concerns | Major concerns | High |
| SIN:TwRE | No concerns | Low risk | No concerns | Major concerns | No concerns | Major concerns | High |
| baicalin:SIN | Some concerns | Low risk | No concerns | Major concerns | No concerns | Major concerns | High |
| curcumin:SIN | Some concerns | Low risk | Some concerns | Major concerns | No concerns | Major concerns | High |
| olive extract:SIN | Some concerns | Low risk | No concerns | Major concerns | No concerns | Major concerns | High |
| placebo:SIN | Some concerns | Low risk | No concerns | Major concerns | No concerns | Major concerns | High |
| pomegranate extract:SIN | Some concerns | Low risk | No concerns | Major concerns | No concerns | Major concerns | High |
| puerarin:SIN | Some concerns | Low risk | No concerns | Major concerns | No concerns | Major concerns | High |
| quercetin:SIN | Some concerns | Low risk | No concerns | Major concerns | No concerns | Major concerns | High |
| resveratrol:SIN | Some concerns | Low risk | No concerns | Major concerns | No concerns | Major concerns | High |
| sesamin:SIN | Some concerns | Low risk | Some concerns | Major concerns | No concerns | Major concerns | High |
| TGP:TwRE | Some concerns | Low risk | No concerns | Major concerns | No concerns | Major concerns | High |
| baicalin:TGP | Some concerns | Low risk | No concerns | Major concerns | No concerns | Major concerns | High |
| curcumin:TGP | Some concerns | Low risk | No concerns | Major concerns | No concerns | Major concerns | High |
| olive extract:TGP | Major concerns | Low risk | No concerns | Major concerns | No concerns | Major concerns | High |
| pomegranate extract:TGP | Some concerns | Low risk | No concerns | Major concerns | No concerns | Major concerns | High |
| puerarin:TGP | Some concerns | Low risk | No concerns | Major concerns | No concerns | Major concerns | High |
| quercetin:TGP | Major concerns | Low risk | No concerns | Major concerns | No concerns | Major concerns | High |
| resveratrol:TGP | Major concerns | Low risk | No concerns | Major concerns | No concerns | Major concerns | High |
| sesamin:TGP | Some concerns | Low risk | Some concerns | Major concerns | No concerns | Major concerns | High |
| TGP:usual treatment | Some concerns | Low risk | No concerns | Major concerns | No concerns | Major concerns | High |
| baicalin:TwRE | Some concerns | Low risk | No concerns | Major concerns | No concerns | Major concerns | High |
| curcumin:TwRE | Some concerns | Low risk | Some concerns | Major concerns | No concerns | Major concerns | High |
| olive extract:TwRE | Some concerns | Low risk | No concerns | Major concerns | No concerns | Major concerns | High |
| placebo:TwRE | Some concerns | Low risk | No concerns | Major concerns | No concerns | Major concerns | High |
| pomegranate extract:TwRE | Some concerns | Low risk | No concerns | Major concerns | No concerns | Major concerns | High |
| puerarin:TwRE | Some concerns | Low risk | No concerns | Major concerns | No concerns | Major concerns | High |
| quercetin:TwRE | Some concerns | Low risk | No concerns | Major concerns | No concerns | Major concerns | High |
| resveratrol:TwRE | Some concerns | Low risk | No concerns | Major concerns | No concerns | Major concerns | High |
| sesamin:TwRE | Some concerns | Low risk | Some concerns | Major concerns | No concerns | Major concerns | High |
| baicalin:curcumin | No concerns | Low risk | No concerns | Major concerns | No concerns | Major concerns | High |
| baicalin:olive extract | Some concerns | Low risk | No concerns | Major concerns | No concerns | Major concerns | High |
| baicalin:pomegranate extract | No concerns | Low risk | No concerns | Major concerns | No concerns | Major concerns | High |
| baicalin:puerarin | Some concerns | Low risk | No concerns | Major concerns | No concerns | Major concerns | High |
| baicalin:quercetin | Some concerns | Low risk | No concerns | Major concerns | No concerns | Major concerns | High |
| baicalin:resveratrol | Some concerns | Low risk | No concerns | Major concerns | No concerns | Major concerns | High |
| baicalin:sesamin | No concerns | Low risk | Some concerns | Major concerns | No concerns | Major concerns | High |
| baicalin:usual treatment | Some concerns | Low risk | No concerns | Major concerns | No concerns | Major concerns | High |
| curcumin:olive extract | Some concerns | Low risk | No concerns | Major concerns | No concerns | Major concerns | High |
| curcumin:pomegranate extract | No concerns | Low risk | No concerns | Major concerns | No concerns | Major concerns | High |
| curcumin:puerarin | Some concerns | Low risk | No concerns | Major concerns | No concerns | Major concerns | High |
| curcumin:quercetin | Some concerns | Low risk | No concerns | Major concerns | No concerns | Major concerns | High |
| curcumin:resveratrol | Some concerns | Low risk | No concerns | Major concerns | No concerns | Major concerns | High |
| curcumin:sesamin | No concerns | Low risk | No concerns | Major concerns | No concerns | Major concerns | High |
| olive extract:pomegranate extract | Some concerns | Low risk | No concerns | Major concerns | No concerns | Major concerns | High |
| olive extract:puerarin | Major concerns | Low risk | No concerns | Major concerns | No concerns | Major concerns | High |
| olive extract:quercetin | Major concerns | Low risk | No concerns | Major concerns | No concerns | Major concerns | High |
| olive extract:resveratrol | Major concerns | Low risk | No concerns | Major concerns | No concerns | Major concerns | High |
| olive extract:sesamin | Some concerns | Low risk | Some concerns | Major concerns | No concerns | Major concerns | High |
| olive extract:usual treatment | Some concerns | Low risk | No concerns | Major concerns | No concerns | Major concerns | High |
| placebo:usual treatment | Some concerns | Low risk | No concerns | Major concerns | No concerns | Major concerns | High |
| pomegranate extract:puerarin | Some concerns | Low risk | No concerns | Major concerns | No concerns | Major concerns | High |
| pomegranate extract:quercetin | Some concerns | Low risk | No concerns | Major concerns | No concerns | Major concerns | High |
| pomegranate extract:resveratrol | Some concerns | Low risk | No concerns | Major concerns | No concerns | Major concerns | High |
| pomegranate extract:sesamin | No concerns | Low risk | Some concerns | Major concerns | No concerns | Major concerns | High |
| pomegranate extract:usual treatment | Some concerns | Low risk | No concerns | Major concerns | No concerns | Major concerns | High |
| puerarin:quercetin | Major concerns | Low risk | No concerns | Major concerns | No concerns | Major concerns | High |
| puerarin:resveratrol | Major concerns | Low risk | No concerns | Major concerns | No concerns | Major concerns | High |
| puerarin:sesamin | Some concerns | Low risk | Some concerns | Major concerns | No concerns | Major concerns | High |
| puerarin:usual treatment | Some concerns | Low risk | No concerns | Major concerns | No concerns | Major concerns | High |
| quercetin:resveratrol | Major concerns | Low risk | No concerns | Major concerns | No concerns | Major concerns | High |
| quercetin:sesamin | Some concerns | Low risk | Some concerns | Major concerns | No concerns | Major concerns | High |
| quercetin:usual treatment | Some concerns | Low risk | No concerns | Major concerns | No concerns | Major concerns | High |
| resveratrol:sesamin | Some concerns | Low risk | Some concerns | Major concerns | No concerns | Major concerns | High |
| resveratrol:usual treatment | Some concerns | Low risk | No concerns | Major concerns | No concerns | Major concerns | High |
| sesamin:usual treatment | Some concerns | Low risk | Some concerns | Major concerns | No concerns | Major concerns | High |

**Table 4. CINeMA Results of DAS28**

| Comparison | Within-study bias | Reporting bias | Indirectness | Imprecision | Heterogeneity | Incoherence | Confidence rating |
| --- | --- | --- | --- | --- | --- | --- | --- |
| SIN:usual treatment | No concerns | Low risk | No concerns | Major concerns | No concerns | Major concerns | High |
| placebo:TGP | Some concerns | Low risk | No concerns | Major concerns | No concerns | Major concerns | High |
| TwRE:usual treatment | No concerns | Low risk | No concerns | Major concerns | No concerns | Major concerns | High |
| curcumin:placebo | No concerns | Low risk | No concerns | No concerns | Major concerns | Major concerns | High |
| curcumin:usual treatment | Major concerns | Low risk | Some concerns | Major concerns | No concerns | Major concerns | High |
| placebo:pomegranate extract | No concerns | Low risk | No concerns | Major concerns | No concerns | Major concerns | High |
| placebo:puerarin | Some concerns | Low risk | No concerns | Major concerns | No concerns | Major concerns | High |
| placebo:quercetin | Major concerns | Low risk | No concerns | Major concerns | No concerns | Major concerns | High |
| placebo:resveratrol | Major concerns | Low risk | No concerns | No concerns | Major concerns | Major concerns | High |
| placebo:sesamin | No concerns | Low risk | Some concerns | Major concerns | No concerns | Major concerns | High |
| SIN:TGP | Some concerns | Low risk | No concerns | Major concerns | No concerns | Major concerns | High |
| SIN:TwRE | No concerns | Low risk | No concerns | Major concerns | No concerns | Major concerns | High |
| curcumin:SIN | Some concerns | Low risk | Some concerns | Major concerns | No concerns | Major concerns | High |
| placebo:SIN | Some concerns | Low risk | No concerns | Major concerns | No concerns | Major concerns | High |
| pomegranate extract:SIN | Some concerns | Low risk | No concerns | Major concerns | No concerns | Major concerns | High |
| puerarin:SIN | Some concerns | Low risk | No concerns | Major concerns | No concerns | Major concerns | High |
| quercetin:SIN | Some concerns | Low risk | No concerns | Major concerns | No concerns | Major concerns | High |
| resveratrol:SIN | Some concerns | Low risk | No concerns | Major concerns | No concerns | Major concerns | High |
| sesamin:SIN | Some concerns | Low risk | Some concerns | Major concerns | No concerns | Major concerns | High |
| TGP:TwRE | Some concerns | Low risk | No concerns | Major concerns | No concerns | Major concerns | High |
| curcumin:TGP | Some concerns | Low risk | No concerns | No concerns | Major concerns | Major concerns | High |
| pomegranate extract:TGP | Some concerns | Low risk | No concerns | Major concerns | No concerns | Major concerns | High |
| puerarin:TGP | Some concerns | Low risk | No concerns | Major concerns | No concerns | Major concerns | High |
| quercetin:TGP | Major concerns | Low risk | No concerns | Major concerns | No concerns | Major concerns | High |
| resveratrol:TGP | Major concerns | Low risk | No concerns | Major concerns | No concerns | Major concerns | High |
| sesamin:TGP | Some concerns | Low risk | Some concerns | Major concerns | No concerns | Major concerns | High |
| TGP:usual treatment | Some concerns | Low risk | No concerns | Major concerns | No concerns | Major concerns | High |
| curcumin:TwRE | Some concerns | Low risk | Some concerns | Major concerns | No concerns | Major concerns | High |
| placebo:TwRE | Some concerns | Low risk | No concerns | Major concerns | No concerns | Major concerns | High |
| pomegranate extract:TwRE | Some concerns | Low risk | No concerns | Major concerns | No concerns | Major concerns | High |
| puerarin:TwRE | Some concerns | Low risk | No concerns | Major concerns | No concerns | Major concerns | High |
| quercetin:TwRE | Some concerns | Low risk | No concerns | Major concerns | No concerns | Major concerns | High |
| resveratrol:TwRE | Some concerns | Low risk | No concerns | Major concerns | No concerns | Major concerns | High |
| sesamin:TwRE | Some concerns | Low risk | Some concerns | Major concerns | No concerns | Major concerns | High |
| curcumin:pomegranate extract | No concerns | Low risk | No concerns | Major concerns | No concerns | Major concerns | High |
| curcumin:puerarin | Some concerns | Low risk | No concerns | Major concerns | No concerns | Major concerns | High |
| curcumin:quercetin | Some concerns | Low risk | No concerns | Major concerns | No concerns | Major concerns | High |
| curcumin:resveratrol | Some concerns | Low risk | No concerns | Major concerns | No concerns | Major concerns | High |
| curcumin:sesamin | No concerns | Low risk | Some concerns | Major concerns | No concerns | Major concerns | High |
| placebo:usual treatment | Some concerns | Low risk | Some concerns | Major concerns | No concerns | Major concerns | High |
| pomegranate extract:puerarin | Some concerns | Low risk | No concerns | Major concerns | No concerns | Major concerns | High |
| pomegranate extract:quercetin | Some concerns | Low risk | No concerns | Major concerns | No concerns | Major concerns | High |
| pomegranate extract:resveratrol | Some concerns | Low risk | No concerns | Major concerns | No concerns | Major concerns | High |
| pomegranate extract:sesamin | No concerns | Low risk | Some concerns | Major concerns | No concerns | Major concerns | High |
| pomegranate extract:usual treatment | Some concerns | Low risk | No concerns | Major concerns | No concerns | Major concerns | High |
| puerarin:quercetin | Major concerns | Low risk | No concerns | Major concerns | No concerns | Major concerns | High |
| puerarin:resveratrol | Major concerns | Low risk | No concerns | Major concerns | No concerns | Major concerns | High |
| puerarin:sesamin | Some concerns | Low risk | Some concerns | Major concerns | No concerns | Major concerns | High |
| puerarin:usual treatment | Some concerns | Low risk | No concerns | Major concerns | No concerns | Major concerns | High |
| quercetin:resveratrol | Major concerns | Low risk | No concerns | Major concerns | No concerns | Major concerns | High |
| quercetin:sesamin | Some concerns | Low risk | Some concerns | Major concerns | No concerns | Major concerns | High |
| quercetin:usual treatment | Some concerns | Low risk | No concerns | Major concerns | No concerns | Major concerns | High |
| resveratrol:sesamin | Some concerns | Low risk | Some concerns | Major concerns | No concerns | Major concerns | High |
| resveratrol:usual treatment | Some concerns | Low risk | No concerns | Major concerns | No concerns | Major concerns | High |
| sesamin:usual treatment | Some concerns | Low risk | Some concerns | Major concerns | No concerns | Major concerns | High |

**Table 5. CINeMA Results of SJC**

| Comparison | Within-study bias | Reporting bias | Indirectness | Imprecision | Heterogeneity | Incoherence | Confidence rating |
| --- | --- | --- | --- | --- | --- | --- | --- |
| SIN:usual treatment | No concerns | Low risk | No concerns | Major concerns | No concerns | Major concerns | High |
| placebo:TGP | Some concerns | Low risk | No concerns | Major concerns | No concerns | Major concerns | High |
| TwRE:usual treatment | Some concerns | Low risk | No concerns | Major concerns | No concerns | Major concerns | High |
| curcumin:placebo | No concerns | Low risk | No concerns | No concerns | Major concerns | Major concerns | High |
| curcumin:usual treatment | Major concerns | Low risk | Some concerns | Major concerns | No concerns | Major concerns | High |
| placebo:pomegranate extract | No concerns | Low risk | No concerns | Major concerns | No concerns | Major concerns | High |
| placebo:quercetin | Major concerns | Low risk | No concerns | Major concerns | No concerns | Major concerns | High |
| placebo:resveratrol | Major concerns | Low risk | No concerns | Major concerns | No concerns | Major concerns | High |
| placebo:sesamin | No concerns | Low risk | Some concerns | Major concerns | No concerns | Major concerns | High |
| SIN:TGP | Some concerns | Low risk | No concerns | Major concerns | No concerns | Major concerns | High |
| SIN:TwRE | No concerns | Low risk | No concerns | Major concerns | No concerns | Major concerns | High |
| curcumin:SIN | Some concerns | Low risk | Some concerns | Major concerns | No concerns | Major concerns | High |
| placebo:SIN | Some concerns | Low risk | No concerns | Major concerns | No concerns | Major concerns | High |
| pomegranate extract:SIN | Some concerns | Low risk | No concerns | Major concerns | No concerns | Major concerns | High |
| quercetin:SIN | Some concerns | Low risk | No concerns | Major concerns | No concerns | Major concerns | High |
| resveratrol:SIN | Some concerns | Low risk | No concerns | Major concerns | No concerns | Major concerns | High |
| sesamin:SIN | Some concerns | Low risk | Some concerns | Major concerns | No concerns | Major concerns | High |
| TGP:TwRE | Some concerns | Low risk | No concerns | Major concerns | No concerns | Major concerns | High |
| curcumin:TGP | Some concerns | Low risk | No concerns | Major concerns | No concerns | Major concerns | High |
| pomegranate extract:TGP | Some concerns | Low risk | No concerns | Major concerns | No concerns | Major concerns | High |
| quercetin:TGP | Major concerns | Low risk | No concerns | Major concerns | No concerns | Major concerns | High |
| resveratrol:TGP | Major concerns | Low risk | No concerns | Major concerns | No concerns | Major concerns | High |
| sesamin:TGP | Some concerns | Low risk | Some concerns | Major concerns | No concerns | Major concerns | High |
| TGP:usual treatment | Some concerns | Low risk | No concerns | Major concerns | No concerns | Major concerns | High |
| curcumin:TwRE | Some concerns | Low risk | Some concerns | Major concerns | No concerns | Major concerns | High |
| placebo:TwRE | Some concerns | Low risk | No concerns | Major concerns | No concerns | Major concerns | High |
| pomegranate extract:TwRE | Some concerns | Low risk | No concerns | Major concerns | No concerns | Major concerns | High |
| quercetin:TwRE | Some concerns | Low risk | No concerns | Major concerns | No concerns | Major concerns | High |
| resveratrol:TwRE | Some concerns | Low risk | No concerns | Major concerns | No concerns | Major concerns | High |
| sesamin:TwRE | Some concerns | Low risk | Some concerns | Major concerns | No concerns | Major concerns | High |
| curcumin:pomegranate extract | No concerns | Low risk | No concerns | Major concerns | No concerns | Major concerns | High |
| curcumin:quercetin | Some concerns | Low risk | No concerns | Major concerns | No concerns | Major concerns | High |
| curcumin:resveratrol | Some concerns | Low risk | No concerns | Major concerns | No concerns | Major concerns | High |
| curcumin:sesamin | No concerns | Low risk | Some concerns | Major concerns | No concerns | Major concerns | High |
| placebo:usual treatment | Some concerns | Low risk | Some concerns | Major concerns | No concerns | Major concerns | High |
| pomegranate extract:quercetin | Some concerns | Low risk | No concerns | Major concerns | No concerns | Major concerns | High |
| pomegranate extract:resveratrol | Some concerns | Low risk | No concerns | Major concerns | No concerns | Major concerns | High |
| pomegranate extract:sesamin | No concerns | Low risk | Some concerns | Major concerns | No concerns | Major concerns | High |
| pomegranate extract:usual treatment | Some concerns | Low risk | No concerns | Major concerns | No concerns | Major concerns | High |
| quercetin:resveratrol | Major concerns | Low risk | No concerns | Major concerns | No concerns | Major concerns | High |
| quercetin:sesamin | Some concerns | Low risk | Some concerns | Major concerns | No concerns | Major concerns | High |
| quercetin:usual treatment | Some concerns | Low risk | No concerns | Major concerns | No concerns | Major concerns | High |
| resveratrol:sesamin | Some concerns | Low risk | Some concerns | Major concerns | No concerns | Major concerns | High |
| resveratrol:usual treatment | Some concerns | Low risk | No concerns | Major concerns | No concerns | Major concerns | High |
| sesamin:usual treatment | Some concerns | Low risk | Some concerns | Major concerns | No concerns | Major concerns | High |

**Table 6. CINeMA Results of TJC**

| Comparison | Within-study bias | Reporting bias | Indirectness | Imprecision | Heterogeneity | Incoherence | Confidence rating |
| --- | --- | --- | --- | --- | --- | --- | --- |
| SIN:usual treatment | No concerns | Low risk | No concerns | Major concerns | No concerns | Major concerns | High |
| placebo:TGP | Some concerns | Low risk | No concerns | Major concerns | No concerns | Major concerns | High |
| TwRE:usual treatment | Some concerns | Low risk | No concerns | Major concerns | No concerns | Major concerns | High |
| curcumin:placebo | No concerns | Low risk | No concerns | No concerns | Major concerns | Major concerns | High |
| curcumin:usual treatment | Major concerns | Low risk | Some concerns | Major concerns | No concerns | Major concerns | High |
| placebo:pomegranate extract | No concerns | Low risk | No concerns | Major concerns | No concerns | Major concerns | High |
| placebo:quercetin | Major concerns | Low risk | No concerns | Major concerns | No concerns | Major concerns | High |
| placebo:resveratrol | Major concerns | Low risk | No concerns | Major concerns | No concerns | Major concerns | High |
| placebo:sesamin | No concerns | Low risk | Some concerns | Major concerns | No concerns | Major concerns | High |
| SIN:TGP | Some concerns | Low risk | No concerns | Major concerns | No concerns | Major concerns | High |
| SIN:TwRE | No concerns | Low risk | No concerns | Major concerns | No concerns | Major concerns | High |
| curcumin:SIN | Some concerns | Low risk | Some concerns | Major concerns | No concerns | Major concerns | High |
| placebo:SIN | Some concerns | Low risk | No concerns | Major concerns | No concerns | Major concerns | High |
| pomegranate extract:SIN | Some concerns | Low risk | No concerns | Major concerns | No concerns | Major concerns | High |
| quercetin:SIN | Some concerns | Low risk | No concerns | Major concerns | No concerns | Major concerns | High |
| resveratrol:SIN | Some concerns | Low risk | No concerns | Major concerns | No concerns | Major concerns | High |
| sesamin:SIN | Some concerns | Low risk | No concerns | Major concerns | No concerns | Major concerns | High |
| TGP:TwRE | Some concerns | Low risk | No concerns | Major concerns | No concerns | Major concerns | High |
| curcumin:TGP | Some concerns | Low risk | No concerns | Major concerns | No concerns | Major concerns | High |
| pomegranate extract:TGP | Some concerns | Low risk | No concerns | Major concerns | No concerns | Major concerns | High |
| quercetin:TGP | Major concerns | Low risk | No concerns | Major concerns | No concerns | Major concerns | High |
| resveratrol:TGP | Major concerns | Low risk | No concerns | Major concerns | No concerns | Major concerns | High |
| sesamin:TGP | Some concerns | Low risk | Some concerns | Major concerns | No concerns | Major concerns | High |
| TGP:usual treatment | Some concerns | Low risk | No concerns | Major concerns | No concerns | Major concerns | High |
| curcumin:TwRE | Some concerns | Low risk | Some concerns | Major concerns | No concerns | Major concerns | High |
| placebo:TwRE | Some concerns | Low risk | No concerns | Major concerns | No concerns | Major concerns | High |
| pomegranate extract:TwRE | Some concerns | Low risk | No concerns | Major concerns | No concerns | Major concerns | High |
| quercetin:TwRE | Some concerns | Low risk | No concerns | Major concerns | No concerns | Major concerns | High |
| resveratrol:TwRE | Some concerns | Low risk | No concerns | Major concerns | No concerns | Major concerns | High |
| sesamin:TwRE | Some concerns | Low risk | No concerns | Major concerns | No concerns | Major concerns | High |
| curcumin:pomegranate extract | No concerns | Low risk | No concerns | Major concerns | No concerns | Major concerns | High |
| curcumin:quercetin | Some concerns | Low risk | No concerns | Major concerns | No concerns | Major concerns | High |
| curcumin:resveratrol | Some concerns | Low risk | No concerns | Major concerns | No concerns | Major concerns | High |
| curcumin:sesamin | No concerns | Low risk | Some concerns | Major concerns | No concerns | Major concerns | High |
| placebo:usual treatment | Some concerns | Low risk | Some concerns | Major concerns | No concerns | Major concerns | High |
| pomegranate extract:quercetin | Some concerns | Low risk | No concerns | Major concerns | No concerns | Major concerns | High |
| pomegranate extract:resveratrol | Some concerns | Low risk | No concerns | Major concerns | No concerns | Major concerns | High |
| pomegranate extract:sesamin | No concerns | Low risk | Some concerns | Major concerns | No concerns | Major concerns | High |
| pomegranate extract:usual treatment | Some concerns | Low risk | No concerns | Major concerns | No concerns | Major concerns | High |
| quercetin:resveratrol | Major concerns | Low risk | No concerns | Major concerns | No concerns | Major concerns | High |
| quercetin:sesamin | Some concerns | Low risk | Some concerns | Major concerns | No concerns | Major concerns | High |
| quercetin:usual treatment | Some concerns | Low risk | No concerns | Major concerns | No concerns | Major concerns | High |
| resveratrol:sesamin | Some concerns | Low risk | Some concerns | Major concerns | No concerns | Major concerns | High |
| resveratrol:usual treatment | Some concerns | Low risk | No concerns | Major concerns | No concerns | Major concerns | High |
| sesamin:usual treatment | Some concerns | Low risk | Some concerns | Major concerns | No concerns | Major concerns | High |

**Table 7. Consistency test for VAS**

|  | Coef | Std.Err | z | P>\|z\| | [95% Conf. Interval] | |
| --- | --- | --- | --- | --- | --- | --- |
| B VS CON | -4.40426 | 17.21374 | -0.26 | 0.798 | -38.14257 | 29.33405 |
| C VS CON | -6.245919 | 18.97977 | -0.33 | 0.742 | -43.44559 | 30.95375 |
| D VS CON | 9.879903 | 20.2737 | 0.49 | 0.626 | -29.85582 | 49.61562 |
| E VS CON | 2.076461 | 25.88988 | 0.08 | 0.936 | -48.66677 | 52.81969 |
| F VS CON | -9.023539 | 25.7742 | -0.35 | 0.726 | -59.54005 | 41.49297 |
| G VS CON | -1.493539 | 25.24117 | -0.06 | 0.953 | -50.96532 | 47.97824 |
| H VS CON | 5.598116 | 13.7601 | 0.41 | 0.684 | -21.37119 | 32.56742 |

**Table 8. Consistency test for inflammatory marker**

|  | Coef | Std.Err | z | P>\|z\| | [95% Conf. Interval] | |
| --- | --- | --- | --- | --- | --- | --- |
| B VS CON | 18.38233 | 33.28865 | 0.55 | 0.581 | -46.86222 | 83.62688 |
| C VS CON | -4.244127 | 33.14213 | -0.13 | 0.898 | -69.20152 | 60.71326 |
| D VS CON | 13.60884 | 40.422 | 0.34 | 0.736 | -65.61683 | 92.8345 |
| E VS CON | -10.77861 | 25.86647 | -0.42 | 0.677 | -61.47596 | 39.91874 |
| F VS CON | 18.90485 | 40.49047 | 0.47 | 0.641 | -60.45501 | 98.26471 |
| G VS CON | 16.57043 | 25.14782 | 0.66 | 0.510 | -32.71839 | 65.85926 |
| H VS CON | 6.358053 | 41.21708 | 0.15 | 0.877 | -74.42595 | 87.14205 |
| I VS CON | 10.32805 | 40.719 | 0.25 | 0.800 | -69.47971 | 90.13582 |
| J VS CON | 10.65805 | 40.67129 | 0.26 | 0.793 | -69.05622 | 90.37232 |
| K VS CON | -2.441947 | 40.55113 | -0.06 | 0.952 | -81.9207 | 77.0368 |
| L VS CON | 11.17805 | 40.46859 | 0.28 | 0.782 | -68.13893 | 90.49504 |
| M VS CON | -.5525156 | 25.01329 | -0.02 | 0.982 | -49.57766 | 48.47263 |

**Table 9. Consistency test for DAS28**

|  | Coef | Std.Err | z | P>\|z\| | [95% Conf. Interval] | |
| --- | --- | --- | --- | --- | --- | --- |
| B VS CON | 1.34712 | 1.698225 | 0.79 | 0.428 | -1.98134 | 4.67558 |
| C VS CON | .388936 | 1.427224 | 0.03 | 0.978 | -2.758414 | 2.836201 |
| D VS CON | 0.1576445 | 1.468207 | 0.11 | 0.914 | -2.719988 | 3.035277 |
| E VS CON | 1.486869 | 1.54947 | 0.96 | 0.337 | -1.550036 | 4.523774 |
| F VS CON | .6867988 | 1.856389 | 0.37 | 0.711 | -2.951657 | 4.325254 |
| G VS CON | 1.766799 | 1.987843 | 0.89 | 0.374 | -2.129302 | 5.662899 |
| H VS CON | 1.026799 | 1.86455 | 0.55 | 0.582 | -2.627653 | 4.68125 |
| I VS CON | -.1732012 | 1.836644 | -0.09 | 0.925 | -3.772957 | 3.426555 |
| J VS CON | .6067988 | 1.883078 | 0.32 | 0.747 | -3.083966 | 4.297563 |
| K VS CON | .4988958 | 1.003312 | 0.50 | 0.619 | -1.46756 | 2.465352 |

**Table 10. Consistency test for SJC**

|  | Coef | Std.Err | z | P>\|z\| | [95% Conf. Interval] | |
| --- | --- | --- | --- | --- | --- | --- |
| B VS CON | 4.387811 | 5.671229 | 0.77 | 0.439 | -6.727594 | 15.50322 |
| C VS CON | .5160745 | 3.886619 | 0.13 | 0.894 | -7.10156 | 8.133709 |
| D VS CON | -.0155573 | 4.4681 | -0.00 | 0.997 | -8.772872 | 8.741757 |
| E VS CON | 4.389983 | 4.731386 | 0.93 | 0.353 | -4.883363 | 13.66333 |
| F VS CON | 3.007856 | 5.726181 | 0.53 | 0.599 | -8.215252 | 14.23096 |
| G VS CON | 4.387856 | 5.67217 | 0.77 | 0.439 | -6.729394 | 15.50511 |
| H VS CON | 2.787856 | 5.668407 | 0.49 | 0.623 | -8.322018 | 13.89773 |
| I VS CON | 2.177856 | 5.833822 | 0.37 | 0.709 | -9.256225 | 13.61194 |
| J VS CON | 1.476744 | 3.169259 | 0.47 | 0.641 | -4.73489 | 7.688378 |

**Table 11. Consistency test for TJC**

|  | Coef | Std.Err | z | P>\|z\| | [95% Conf. Interval] | |
| --- | --- | --- | --- | --- | --- | --- |
| B VS CON | 3.970407 | 5.989584 | 0.66 | 0.507 | -7.768962 | 15.70978 |
| C VS CON | -.9173652 | 4.070002 | -0.23 | 0.822 | -8.894422 | 7.059692 |
| D VS CON | -1.552016 | 4.742479 | -0.33 | 0.743 | -10.8471 | 7.743073 |
| E VS CON | 3.472596 | 5.011895 | 0.69 | 0.488 | -6.350537 | 13.29573 |
| F VS CON | -.7292316 | 6.292071 | -0.12 | 0.908 | -13.06146 | 11.603 |
| G VS CON | 2.970768 | 5.980373 | 0.50 | 0.619 | -8.750548 | 14.69208 |
| H VS CON | .8707684 | 5.981134 | 0.15 | 0.884 | -10.85204 | 12.59358 |
| I VS CON | .9007684 | 6.41354 | 0.14 | 0.888 | -11.66954 | 13.47108 |
| J VS CON | .990949 | 3.290032 | 0.30 | 0.763 | -5.457395 | 7.439293 |
